# Development of a deep learning model for cancer diagnosis by inspecting cell-free DNA end-motifs

**DOI:** 10.1038/s41698-024-00635-5

**Published:** 2024-07-27

**Authors:** Hongru Shen, Meng Yang, Jilei Liu, Kexin Chen, Xiangchun Li

**Affiliations:** 1 Tianjin Cancer Institute, Tianjin’s Clinical Research Center for Cancer, National Clinical Research Center for Cancer, Tianjin Medical University Cancer Institute and Hospital, Tianjin Medical University, Tianjin, China; 2 Department of Epidemiology and Biostatistics, Tianjin’s Clinical Research Center for Cancer, Key Laboratory of Molecular Cancer Epidemiology of Tianjin, Key Laboratory of Prevention and Control of Major Diseases in the Population Ministry of Education, National Clinical Research Center for Cancer, Tianjin Medical University Cancer Institute and Hospital, Tianjin Medical University, Tianjin, China

**Keywords:** Cancer, Diagnostic markers

## Abstract

Accurate discrimination between patients with and without cancer from cfDNA is crucial for early cancer diagnosis. Herein, we develop and validate a deep-learning-based model entitled end-motif inspection via transformer (EMIT) for discriminating individuals with and without cancer by learning feature representations from cfDNA end-motifs. EMIT is a self-supervised learning approach that models rankings of cfDNA end-motifs. We include 4606 samples subjected to different types of cfDNA sequencing to develop EIMIT, and subsequently evaluate classification performance of linear projections of EMIT on six datasets and an additional inhouse testing set encopassing whole-genome, whole-genome bisulfite and 5-hydroxymethylcytosine sequencing. The linear projection of representations from EMIT achieved area under the receiver operating curve (AUROC) values ranged from 0.895 (0.835–0.955) to 0.996 (0.994–0.997) across these six datasets, outperforming its baseline by significant margins. Additionally, we showed that linear projection of EMIT representations can achieve an AUROC of 0.962 (0.914–1.0) in identification of lung cancer on an independent testing set subjected to whole-exome sequencing. The findings of this study indicate that a transformer-based deep learning model can learn cancer-discrimative representations from cfDNA end-motifs. The representations of this deep learning model can be exploited for discriminating patients with and without cancer.

## Introduction

Liquid biopsies based on plasma cell-free DNA (cfDNA) are repertoire for emergent markers that have been actively studied for cancer diagnosis^1^. The circulating cfDNA in the plasma is originated mostly from hematopoietic cells and admixed with DNA fragments released from tumor cells due to cell apoptosis and necrosis^2^. There are different topological forms of cfDNA that exist in the plasma, such as single- and double-stranded DNA, linear- and circular-mitochondrial DNA and extrachromosomal circular DNA. The linear form of DNA fragments is the most commonly studied among the other cfDNA topological forms^1^. Size profiling analyses revealed shorter modal size of cfDNA in cancer patients as compared with healthy individuals. Besides, there is also observations of preferential patterns of cfDNA end-motifs in cancer patients versus non-cancerous controls. These differential features bore by cfDNA fragments have thus been widely studied for cancer diagnosis^3–7^.

The size distributions of cfDNA in oncology have been extensively studied^8^. The size of cfDNA is varying around a median size of 167 bp and exhibiting non-random fragmentation patterns^9^. The unique cfDNA patterns manifest the physiological circumstances in cancer. Cristiano and colleagues reported that patients with cancer had an altered fragmentation profiles as compared with heathy individuals whose fragmentation profiles are dominated by the nucleosomal patterns of white blood cells^10^. The size of cfDNA fragments derived from patients with cancer are more variable than that of cfDNA derived from non-cancerous individuals. Based on this observation, Cristiano and colleagues developed a machine learning model termed DELFI for the detection of different cancer types by incorporating genome-wide fragmentation features of cfDNA subjected to low-coverage whole-genome sequencing^10^. They reported a sensitivity of 91% in detecting patients with cancer across seven cancer types including breast, colorectal, lung, ovarian, pancreatic, gastric and bile duct cancers^10^. Mathios and colleagues demonstrated that combining the prediction from DELFI and thoracic low dose computed tomography significantly improves the sensitivity and specificity in the identification of lung cancer^11^. Recently, Foda and colleagues reported a sensitivity of 88% at the 98% specificity in an average population in the detection of patients with liver cancer by using DELFI to incorporate multi-feature of cfDNA fragmentome data^12^. The other types of signals imprinted on cfDNA fragments have been investigated for early cancer detection. Liu and colleagues reported that bisulfite sequencing of cfDNA fragments offers informative methylation patterns for detection of more than 50 cancer types across stages^13^. Klein and colleagues clinically validated that targeted methylation-based profiling of cfDNA enables high specificity and accuracy of early cancer detection^14^. Recently, Jamshidi and colleagues evaluated cfDNA-based cancer detection via interrogating mutation, whole-genome methylation, allelic imbalance, copy number change, fragment endpoint preference and length^15^. They observed that whole-genome methylation-based approach achieved the best performance among the other approaches^15^.

The fragmentation patterns of cfDNA are not random. End-motif profiling of plasma cfDNA has been found to be an emergent fragmentomic marker in hepatocellular carcinoma (HCC). Jiang and colleagues reported that there was discrepancy of end-motifs between patients with HCC and those without HCC^3^. The diversity of plasma cfDNA end-motifs are increased in subjects with HCC. In particular, the 4-nucleotide oligomer (i.e., 4-mer) motifs such as CCCA, CCAG and CCTG are more prevalent in subjects with HCC versus those without HCC^3^. Meanwhile, they observed that liver-derived cfDNA are frequently ended at certain genomic positions than non-liver-derived cfDNA^16^. Patients with hepatocellular carcinoma exhibited different nonrandom distribution of cfDNA at specific genomic coordinates as compared with liver transplant recipients and individuals with hepatitis B^16^. The preferential distribution of cfDNA among the genome can be explored for capturing the evidence of cancer via liquid biopsy. In addition, the jagged-end sequences from plasma and urine were reported to be a novel marker in the diagnosis of cancer. Zhou and colleagues characterized patterns of jagged-end motifs of cfDNA from urine and plasma and designed the jagged-end index for quantitative measurement of end-motifs. They reported jagged-end index of cfDNA can be used to differentiate patients with and without bladder cancer^17^.

In the past three years, self-supervised learning has emerged as an important research direction in artificial intelligence. Self-supervised methods have advantages over supervised methods as the former use the unlabeled datasets whereas the later require manual annotation of each datapoint. Therefore, self-supervised methods can exploit far larger amount of data as compared with supervised methods. The transformer architecture proposed by Vaswani and colleagues in 2017 has emerged as a powerful general-purpose architecture for representation learning, achieving state-of-the-art performance in natural language understanding^18^. High capacity transformer models trained with hundreds of millions of protein sequences capture biological structures and functions of proteins and enable zero-shot prediction of the effects of mutation on protein function^19,20^. Recently, we showed that transformer model trained with gene symbols ordered by expression levels from 10 million single-cell transcriptomes is able to learn robust features representing different cell types^21^.

Our study is motivated by the urgent need to overcome two significant challenges in cfDNA-based cancer diagnosis. Firstly, there is a pressing demand for more accurate and reliable methods for early cancer detection, an area where current diagnostic approaches often prove inadequate. Despite advancements in liquid biopsy techniques, accurately discriminating between individuals with and without cancer based on cfDNA remains a formidable challenge. Secondly, traditional bioinformatics methods for cfDNA analysis involve laborious and error-prone steps such as reads mapping, detection of copy number changes, and analysis of fragmentome characteristics. This complex pipeline not only increases the risk of errors but also poses a significant barrier to widespread adoption due to its intricacy. Our study aims to address these challenges by investigating a deep-learning-based end-to-end approach that streamlines the cfDNA analysis process, offering ease of use while enhancing accuracy and reliability.

In this study, we proposed a deep-learning-based approach – end-motif inspection via transformer (EMIT) – that models the rankings of end-motif frequencies of cfDNA fragments. We developed EMIT with a total number of 4606 plasma cfDNA samples subjected to genome sequencing, bisulfite sequencing and 5-hydroxymethylcytosine sequencing. We observed that cancer-discriminative features are encoded and represented within EMIT even though only end-motif frequencies but not information of cancer states are used in the development of EMIT. Linear projection of representations extracted from EMIT could achieve high classification performance in the identification of cancer on 6 datasets generated by different kind of sequencing types. In addition, we demonstrated high classification performance in the identification of lung cancer from linear projections of EMIT representations on an independent cfDNA testing set subjected to whole-exome sequencing.

## Results

### An overview of EMIT

**Fig. 1:**
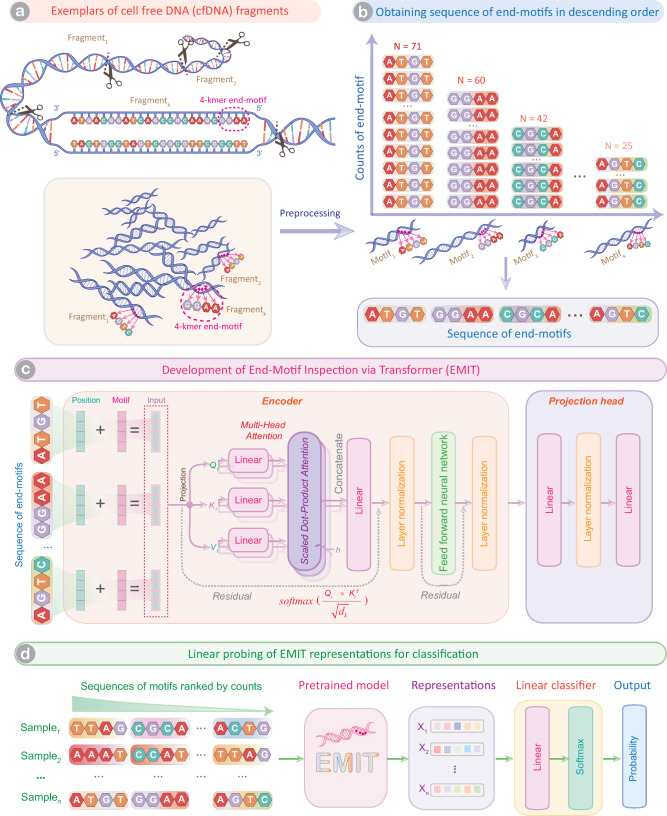
A flowchart depicting the development and validation of EMIT. **a** An exemplar illustrating cfDNA and its 4-mer end-motif. **b** Construction of end-motif sequence from sequencing reads. **c** The archietecture of EMIT. **d** Linear projection of end-motif sequence representations from EMIT for diagnosis of cancer.

EMIT is a transformer architecture with projection head that models the distributions of end-motifs by incorporating context from across the sequence. We preprocessed the raw sequencing reads to obtain the input for EMIT (Fig. 1a). We calculated the frequencies of different end-motifs and sorted them in a descending order to obtain sequence of end-motifs (Fig. 1b). EMIT takes this sorted sequence of end-motifs as the input. EMIT consists of an embedding layer followed by a transformer encoder and projection layer (Fig. 1c). Suppose that we take *k*-nucleotide sequence from the 5’ end of cfDNA fragment as the end-motif (i.e. *k*-mer motif), thus we have a dictionary ***D*** with a total number of 4^*k*^ motifs (See Methods). In this study, we used 4-mer motif by setting *k* = 4. The sequence of end-motifs fed into EMIT is denoted as ***M*** = {CLS, *m*_*0*_, *m*_*1*_, …, *m*_*i*_, …, *m*_*t*_, SEP; *t* < 4^*k*^}. We used 128 end-motifs for developing EMIT. CLS and SEP are two special motifs that are inserted into the beginning and end of input motif sequence. The embedding layer transforms the *i*th motif *m*_*i*_ into a feature vector of *d*-dimension *x*_*i*_, giving rise to a feature matrix of ***X*** = {*x*_*0*_, *x*_*1*_, …, *x*_*i*_…, *x*_*t*_; *t* < 4^*k*^}^T^ for motif sequence ***M***. Besides, the embedding layer embeds the positions of each motif in ***M*** into a feature vector of *d*-dimension, denoted as ***P*** = {*p*_*0*_, *p*_*1*_, …, *p*_*t*_; *t* < 4^*k*^}^T^. The transformer encoder takes the element-wise summation of ***X*** and ***P*** (i.e., ***X*** + ***P***) as input and outputs a representation matrix ***Z***, which has exactly the same dimensions as ***X*** and ***P***. The projection head layer maps ***Z*** into the distributions of each motif within motif dictionary ***D***. The transformer encoder consists of self-attention module followed by position-wise feed-forward neural network. The self-attention explicitly models pair-wise interaction between all motifs in the input sequence, thus the transformer encoder directly represents cooccurrence of motifs (Fig. 1c). Since the CLS aggregates all of the motifs to obtain representation of the whole input sequence, the attention score between motif *m*_*i*_ and CLS (denoted as *a*_*i*_) represents the influence of *m*_*i*_ on the representation of ***M***. We followed the scheme proposed by Devlin and colleagues^22^ to randomly corrupt 15% of each input motif sequence by replacing the chosen motif with [MASK] motif at 80%, random motif at 10% and the same motif at 10%. We trained EMIT using the masked language modeling objective to maximize the log-likelihood of masked motifs rather than the entire motif sequence. Finally, we used the pretrained EMIT model to extract feature representations and perform linear projection of these representations for identification of cancer (Fig. 1d). Linear projection allows for identifying information about the difference between cancer and control that is linearly encoded within the representations from EMIT.

### Evaluation of EMIT models

**Fig. 2:**
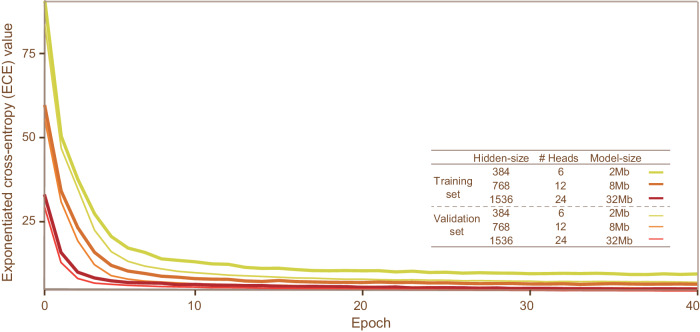
The exponentiated cross-entropy (ECE) values for different parameter sizes of EMIT models. The x-axis is training epochs and y-axis is ECE values.

We collected a total number of 4606 samples from public database for the development of EMIT models (See Methods). We derived 204,366 sequences of end-motifs from these samples (See Methods). We trained three EMIT models that have different parameter size by varying the hidden size of the transformer encoder. We considered a hidden size of 384, 768 and 1536, giving rise to parameter size of 2, 8 and 32 megabytes, respectively. For the convenience of description, we denoted these model as EMIT-2Mb, EMIT-8Mb and EMIT-32Mb. We hold out a random number of 5000 instances for monitoring the loss of the pretrained model. We used the exponentiated cross entropy (ECE) as the primary metric to compare these models. We trained these models by using Adam optimizer, batch size of 256, weight decay of 0.01 and learning rate of 1e−4 for 40 epochs. Learning rate was warmup for one epoch and decreased towards zero by following cosine scheduling. The ECE value is defined as the exponential of model loss averaged over per end-motif. We observed that bigger model has consistently lower ECE value at each epoch as compared with smaller model (Fig. 2).

### Linear projection of EMIT representations for identification of cancer

To determine whether the information about cancer is encoded and represented within EMIT, we performed linear projection for representations extracted from EMIT for identification of cancer (See Methods). We collected 4606 samples from six datasets covering hepatocellular carcinoma (HCC), colorectal cancer (CRC), lung cancer (LUCA) and esophageal carcinoma (ESCA) to examine the classification performance. These six datasets included plasma cfDNA samples subjected to different types of sequencing such as whole-genome sequencing, whole-genome bisulfite sequencing, targeted bisulfite sequencing and 5-hydroxymethylcytosine sequencing. We considered linear projection of end-motif counts as the baseline method for comparison.

**Fig. 3:**
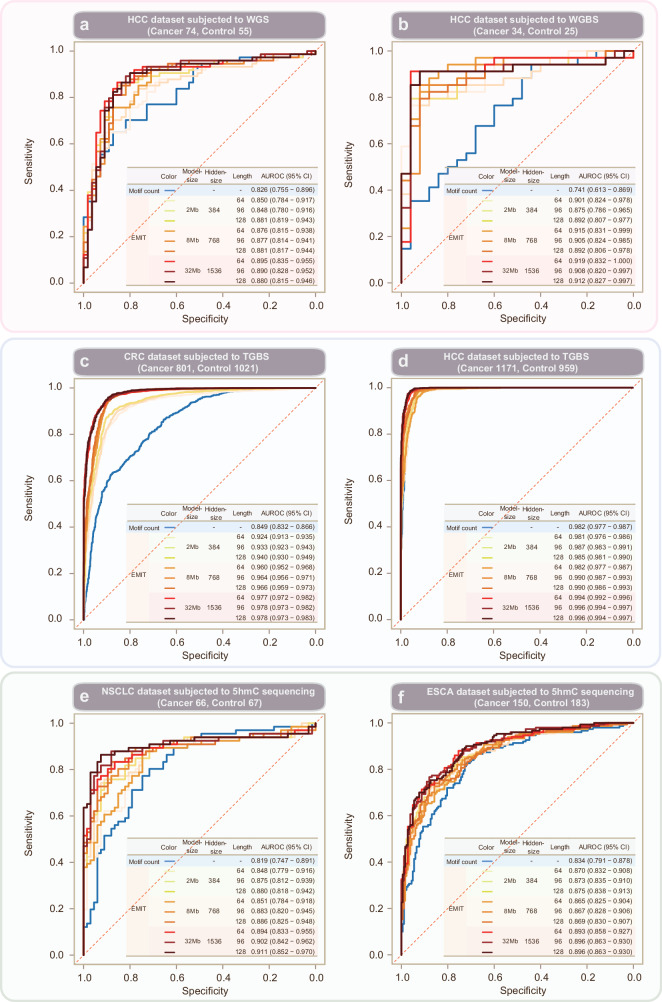
The receiver operating characteristic (ROC) curves of different EMIT models with different input sequence length across six datasets. **a** ROC curves on the hepatocellular carcinoma dataset subjected to whole-genome sequencing. **b** ROC curves on the hepatocellular carcinoma dataset subjected to whole-genome bisulfite sequencing. **c** ROC curves on the colorectal dataset subjected to targeted bisulfite sequencing. **d** ROC curves on the hepatocellular carcinoma dataset subjected to targeted bisulfite sequencing. **e** ROC curves on the lung cancer dataset subjected to 5-hydroxymethylcytosine sequencing. **f** ROC curves on the esophageal carcinoma dataset subjected to 5-hydroxymethylcytosine sequencing.

**Fig. 4:**
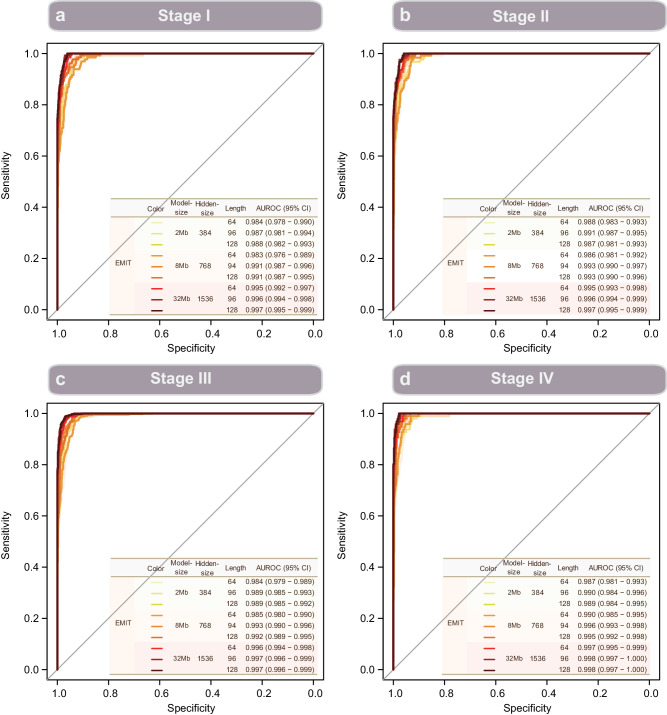
Receiver operating characteristic curves of EMIT models on HCC-WGBS dataset stratified by tumor stages. **s**tage I (**a**), stage II (**b**), **s**tage III (**c**), and **s**tage IV (**d**).

Across these six datasets, representations from EMIT achieved better classification performance as compared with the baseline. Besides, representations from bigger EMIT model has higher classification performance as compared with smaller model (Fig. 3). On the HCC dataset subjected to whole-genome sequencing, the baseline method achieved an AUROC of 0.826 (0.755–0.896) whereas linear projection of representations from EMIT models achieved an AUROC ranged from 0.848 (0.780–0.917) to 0.895 (0.835–0.955) (Fig. 3a). On the HCC dataset subjected to whole-genome bisulfite sequencing, the AUROC values are ranged from 0.875 (0.786–0.965) to 0.919 (0.832–1.0) for representations from EMIT models versus 0.741 (0.613–0.869) for the baseline method (Fig. 3b). With respect to two datasets subjected to targeted bisulfite sequencing for detection of CRC and HCC, the baseline method achieved an AUROC of 0.849 (0.832–0.866) and 0.982 (0.977–0.987), respectively (Fig. 3c, d); whereas linear projections of representations from EMIT models achieved AUROC values ranged from 0.924 (0.913–0.935) to 0.978 (0.973–0.983) and 0.981 (0.976–0.986) to 0.996 (0.994–0.997) (Fig. 3c, d). On the two datasets subjected to 5-hydroxymethylcytosine sequencing for detection of LUCA and ESCA, the respective AUROC values are 0.819 (0.747–0.891) and 0.834 (0.791–0.878) for the baseline method; whereas AUROC values are ranged from 0.848 (0.779–0.916) to 0.911 (0.852–0.970) and 0.865 (0.825–0.904) to 0.896 (0.863–0.930), respectively (Fig. 3e, f). On the HCC-TGBS dataset that had tumor stage available, EMIT achieved high performance for tumor patients at different stages (Fig. 4). In addition, we observed that linear projection performance of EMIT is positively associated with model size. Big model such as EMIT-32Mb generally has higher AUROC value as compared with EMIT-2Mb (Fig. 3). Meanwhile, the performance of linear projection was associated with input sequence length for small model such as EMIT-2Mb, whereas the impact of sequence length is diminished for big model such as EMIT-32Mb (Fig. 3, Supplementary Figs. 1, 2 and Supplymentary Table 1).

On the Cristiano dataset^10^, EMIT achieved an AUROC ranged from 0.946 to 0.969 in classification of cancer versus control (Supplementary Fig. 3). For the classification of multiple cancer types, EMIT achieved high classification performance with different model sizes and input sequence lengths (Supplementary Figs. 4, 5). For instance, for the input length of 128, EMIT-32Mb achieved a micro-averaged one-versus-rest AUROC of 0.901 (0.880–0.928). When stratified by tumor stages, The micro-averaged one-versus-rest AUROC values of EMIT-32Mb are 0.958 for tumor at stage I, 0.946 at stage II, 0.972 at stage III, and 0.969 at stage IV (Supplementary Fig. 6). When stratified by cancer types, EMIT-32Mb achieved AUROC values ranged from 0.822 (0.760–0.883) for lung cancer to 0.951 (0.923–0.979) for pancreatic cancer (Supplementary Fig. 5). The performance of EMIT-2Mb and EMIT-8Mb were shown in Supplementary Figs. 4, 5. We grouped these sample into 4 group according to the quantiles of mean allele frequency (MAF, MAF < 25%, 25% ≤ MAF < 50%, 50% ≤ MAF < 75% and MAF ≥ 75%) averaged for all mutations. EMIT did not exhibit statistically significant difference across these four groups (Table 1), suggesting the stable performance of EMIT with respect to different MAF.

**Table 1:** The confusion matrix of EMIT with different model sizes stratified by mean mutation allele frequency

Group^a^	Length = 64			Length = 96			Length = 128		
	Incorrect predictions	Correct predictions	Fisher’s exact test	Incorrect predictions	Correct predictions	Fisher’s exact test	Incorrect predictions	Correct predictions	Fisher’s exact test
EMIT-2Mb									
G1	6	10	*P* = 0.53	4	12	*P* = 0.47	4	12	*P* = 0.29
G2	3	13		2	14		1	15	
G3	3	12		1	14		1	14	
G4	6	10		4	12		4	12	
EMIT-8Mb									
G1	3	13	*P* = 0.89	2	14	*P* = 1	2	14	*P* = 1
G2	2	14		1	15		1	15	
G3	2	13		1	14		1	14	
G4	4	12		1	15		1	15	
EMIT-32Mb									
G1	4	12	*P* = 0.41	2	14	*P* = 0.49	2	14	*P* = 0.74
G2	1	15		1	15		1	15	
G3	1	14		0	15		0	15	
G4	3	13		3	13		2	14	

^a^The mean mutation allele frequency is grouped based on quantiles of 25%, 50% and 75%. G1: MAF ≤ 25%, G2: 25% < MAF ≤ 50%, G3: 50% < MAF ≤ 75%, G4: MAF > 75%.

Taken together, we demonstrated that information about cancer was encoded and represented within the pretrained EMIT models. Linear projection of representations from EMIT model is able to achieve high classification performance in the identification of cancer. The classification metrics such as accuracy, sensitivity and specificity are provided in Supplementary Tables 2–7. Additionally, we also observed that linear projection of representations from EMIT significantly outperformed the motif diversity score in the identification of cancer (Supplementary Fig. 7, Delong’s test, *P* < 0.001).

### Preferential end-motifs in cancer versus control

For an input sequence, the first-row vectors of self-attention matrices record the attention scores of the end-motifs on the representation of the input sequence (See Methods). As an exemplar, we analyzed the attention scores of end-motifs for the HCC dataset that was subjected to whole-genome sequencing. With respect to the 5th self-attention head, we observed that CGTC and TATT have higher attention scores while TCTG, TCGC and TAGC have lower attention scores in cancer group as compared with control group (two-sided *t*-test, adjusted *p*-value < 0.05; Supplementary Fig. 8a). With respect to the 16th self-attention head, TATT has higher attention score while TCTG, TTAT, TCGC and TAGC have lower scores in cancer group as compared with control group (two-sided *t*-test, adjusted *p*-value < 0.05; Supplementary Fig. 8b). The attention scores stratified by heads were shown in Supplementary Fig. 9. Meanwhile, the end-motif attention networks also exhibited different topology in cancer group versus control group. For example, we observed that a network module characterized by ATTC as the hub node was depleted in control group but apparent in cancer group within the 12th self-attention head (Supplementary Fig. 8c). Besides, we observed a network module characterized by TACC as the hub node is observable in the control group but not in cancer group within the 16th self-attention head (Supplementary Fig. 8d). All the other end-motif attention networks were provided in Supplementary Fig. 10.

### Linear projection of EMIT representations on independent testing set

**Fig. 5:**
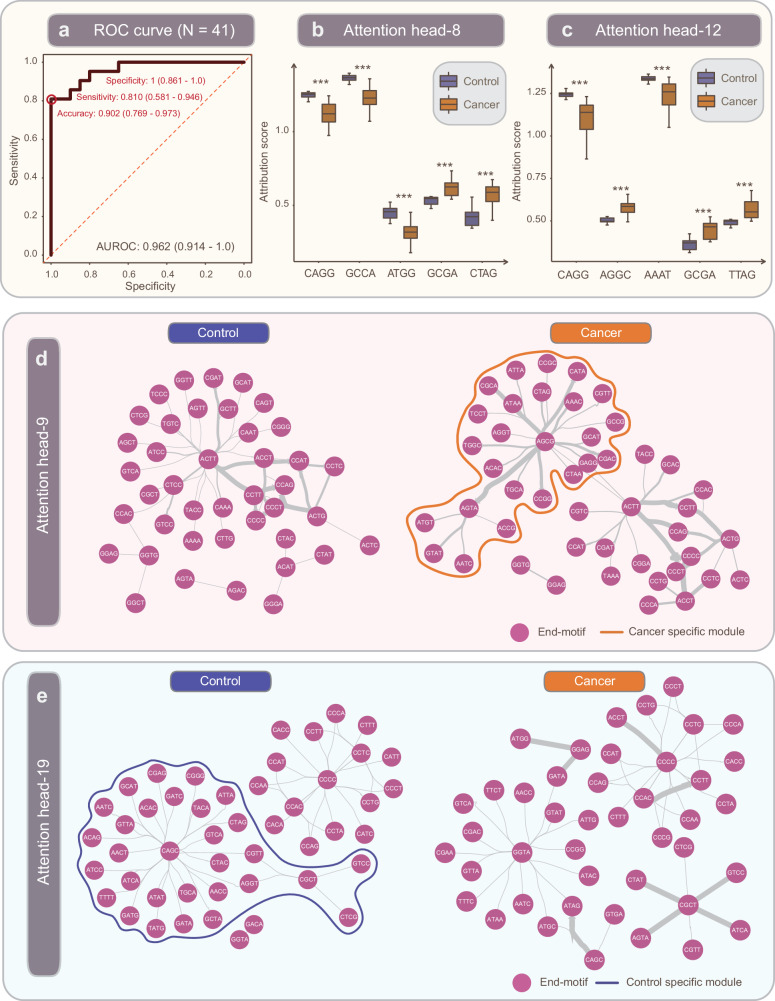
Validation of EMIT on independent inhouse lung cancer dataset. **a** ROC curve of linear projection of representations extracted from EMIT in the diagnosis of cancer. **b**, **c** End-motifs exhibited differential attribution scores. The boxplot represents the distribution of the attribution score, where the box spans the interquartile range (IQR) from the first quartile (Q1) to the third quartile (Q3). The median is denoted by the center line within the box, while the whiskers extend to encompass data points within 1.5 times the IQR from Q1 and Q3. **d**, **e** End-motif attention netoworks exhibited differential topology. *** represented adjusted *p*-value < 0.05.

We trained a linear classifier on EMIT-32Mb representations extracted from all aforementioned 4606 samples and subsequently tested its classification performance on inhouse dataset. This inhouse dataset consisted of 21 patients with lung cancer and 20 non-cancerous controls that were subjected to targeted capture sequencing of plasma cfDNA. We observed that this linear classifier achieved an AUROC of 0.962 (0.914–1.0), accuracy of 0.902 (0.769–0.973), sensitivity of 0.810 (0.581–0.946) and specificity of 1.0 (0.861–1.0) (Fig. 5a). By examining the multi-headed self-attention matrices of EMIT (See Methods), we observed that several end-motifs such as GCGA, CTAG, AGGC, GCGA and TTAG have higher attention scores while end-motifs such as CAGG, GCCA, ATGG, CAGG and AAAT have lower attention scores in cancer group as compared with control group (two-sided *t*-test, adjusted *p*-value < 0.05; Fig. 5b, c). The attention scores stratified by heads were shown in Supplementary Fig. 11. Meanwhile, we also observed that the end-motif networks exhibited different topology in control group versus patients with lung cancer. For example, we observed that network module featured by AGCG as the hub node is not observable in control group but apparent in cancer group (Fig. 5d). A network module with CAGC as the hub node is apparent in control group but not observable in cancer group (Fig. 5e). The other end-motif attention networks were provided in Supplementary Fig. 12.

## Discussion

We proposed a self-supervised method termed EMIT for learning representations of cfDNA end-motifs. We developed EMIT with 4606 plasma cfDNA samples that were subjected to genome sequencing, bisulfite sequencing or 5-hydroxymethylcytosine sequencing. We demonstrated that representations about the difference between cancer and control samples were encoded within the pretrained EMIT models. Linear projections of the representations from EMIT are able to achieve high classification performance in the identification of cancer. EMIT represents a step towards a unified deep-learning approach for characterizing end-motifs of cfDNA fragmentome.

Identification and validation of cancer biomarkers are considered to be one of the challenges to realize early detection of cancer^23^. Assessement of mutations in cfDNA suffers from key limitation in that the number of recurrently cancer-associated mutations is very low^24^. Therefore, new cancer biomarkers are in desperate needed. The frequency of cfDNA end-motifs has been investigated for identification of hepatocellular carcinoma among other liver diseases^3,12^. Certain cfDNA end-motifs are more prevalent than others in association with cancer^3^. These studies used the count values of end-motifs and did not take into account potential association among different end-motifs but treated them independently. In contrast, we explored the nonlinear associations among different end-motifs via transformer algorithm. Indeed, we observed that some certain end-motifs are more frequently cooccurring whereas the others are less frequently cooccurring. For instance, we showed that there are strong associations among different end-motifs.

EMIT has several advantages. It pioneers a new direction for characterization and interpretation of cfDNA end-motifs. EMIT uses a conceptually simple yet empirically powerful approach for representing cfDNA end-motifs in a self-supervised manner, therefore, it can represent a diverse set of cfDNA end-motifs from different sequencing platforms as we demonstrated in this study. EMIT has the potential to provide a unified framework for representing all kinds of cfDNA end-motifs. The intriguing aspect of self-supervised method is that it can assimilate much more data when compared with supervised method as the later requires human-annotated labels whereas the former generates labels from input data. EMIT greatly simplifies the analytical procedures as it only takes the rankings of end-motifs as input, which is computationally efficient to obtain from raw sequencing data. Therefore, the time-consuming steps such as sequence mapping, assessment of copy number changes and detection of mutation are not required. Meanwhile, rankings are insensitive to batch effect as we exmined on the Cristiano dataset^10^ (See Methods; *kBET*^25^ score = 0.854, *P* = 0.298), providing reassurance regarding the robustness of our methodology against batch effects. In addition, we demonstrated that information about cancer is encoded and represented within the pretrained EMIT model and linear projection of the representations extracted from EMIT is able to obtain high classification performance in identification of cancer from different sequencing data types. In this study, we observed that the expressivity of EMIT is associated with its scalability. Big models have lower exponentiated cross-entropy value and higher linear projection performance as compared with small models. The linear projection performance of EMIT is expected to increase as we accumulate more cfDNA sequencing data to train higher capacity of the model.

Batch effect is prevalent in cfDNA-based cancer detection^26,27^. Confounding factors associated with batch effect include genetic variability, environmental and lifestyle factors, sample collection and preparation^26,28^. We have token batch effect into account in our study. We tried to mitigate batch effect via self-supervised pretraining and linear probing for classification. Specifically, self-supervised pretraining has been reported to be less prone towards overfitting and demonstrated to be helpful for improving downstream tasks including classification^29–31^. Linear probing also has the potential to reduce overfitting as it trains a light-weighted linear classifier on the representations extracted from the self-supervised pretraining model^32,33^. The “self-supervised pretraining then linear probing” paradigm can boost generalization and facilitate efficient feature-to-target mapping, while reducing overfitting^33^. While cfDNA in plasma derived from cancer cells is a direct indicator of cancer, we speculate that cfDNA fragments released from non-cancerous cells in patients with cancer might be helpful for cancer detection. Given that cfDNA fragmentome manifests nucleosome topology^1^ and heterogeneous nucleosome organizations^34^ among different type of cells, we hypothesize that the physiological state of patients with cancer can reshape nucleosome topology of the other cell types besides cancer cells, leading to different topological structure of non-cancerous cells versus cancer cells, potentially providing valuable signal for cancer detection. In addition, this dataset was subjected to enrichment of cfDNA fragments according to differential methylation^5^ patterns in cancer tissue versus cancer-adjacent normal control tissue. Therefore, the signal of cancer is greatly amplified. Meanwhile, this dataset^5^ may not present significant challenges, potentially leading to inflated sensitivity and specificity estimates. Taken together, we have the potential to achieve high sensitivity and specificity in detection of cancer at early stage. In-depth future studies are required to investigate and/or verify the aforementioned hypothesis.

Our approach was not without limitations. EMIT expects the rankings of end-motifs as input, therefore, it ignores information such as size profile, aberrant coverage, preferred end coordinates and somatic mutations that have been demonstrated to be helpful for identification of cancer^10^. In addition, the amount of tumor-derived cfDNA is limited especially in patients diagnosed with cancer at early stage. The signal of cancer is massively diluted once material from a cancer gets into the blood and admixes with signals from non-cancerous cells^24^. Enrichment of tumor-derived cfDNA by excluding the background cfDNA according to distribution of size profiles^8^ has the potential to increase tumor signals. Besides, identification of genomic regions that exhibited differential patterns in tumor versus non-cancer control would be beneficial for improvements in identification of cancer. This is exemplified by the high classification performance on the two datasets that were subjected to targeted bisulfite sequencing of specific genomic regions. Incorporation of these evidence is expected to further increase the classification performance of linear projection of EMIT representations in the identification of cancer. We will investigate this potential improvement in our next study.

In summary, we presented a self-supervised method entitled EMIT that can inspect end-motifs of plasma cfDNA subjected to different sequencing techniques simultaneously. We demonstrated that information about cancer is encoded and represented within the pretrained EMIT models. Our method will facilitate characterization of cfDNA end-motifs and translational research of cfDNA.

## Methods

### Data collection

We collected a total number of 4606 samples subjected to different types of cfDNA sequencing from five studies, covering hepatocellular carcinoma (HCC), colorectal cancer (CRC), non-small cell lung cancer (NSCLC) and esophageal carcinoma (ESCA). The sequencing types include whole-genome sequencing (WGS), whole-genome bisulfite sequencing (WGBS), targeted genome bisulfite sequencing (TGBS) and 5- hydroxymethylcytosine sequencing (5hmC).

The HCC-WGS dataset consisted of plasma cfDNA samples subjected to WGS from 74 patients with HCC and 55 controls without HCC. We downloaded it from European Bioinformatics Institute (Accession No. EGAS00001003409)^3^.

The HCC-WGBS dataset consisted of plasma cfDNA samples subjected to WGS from 34 patients with HCC and 25 controls without HCC. Raw sequencing data were applied and downloaded it from European Bioinformatics Institute (Accession No. EGAS00001003409)^3^.

The CRC-TGBS dataset consisted of plasma cfDNA samples subjected to TGBS from 801 patients with CRC and 1021 healthy controls. Raw sequencing data were downloaded from Sequence Read Archive database (Accession No. PRJNA574555)^4^.

The HCC-TGBS dataset consisted of plasma cfDNA samples subjected to TGBS from 1171 patients with HCC and 959 healthy controls. Raw sequencing data were downloaded from Sequence Read Archive (Accession No. PRJNA360288)^5^.

The NSCLC-5hmCS dataset consisted of plasma cfDNA samples subjected to 5hmCS from 66 patients with NSCLC and 67 healthy controls. Raw sequencing data were downloaded from Genome Sequence Archive database (Accession No. PRJCA000816)^7^.

The ESCA-5hmCS dataset consisted of plasma cfDNA samples subjected to 5hmCS from 150 patients with ESCA and 183 healthy controls. Raw sequencing data were downloaded from Genome Sequence Archive database (Accession No. PRJCA000646)^6^.

The Cristiano dataset consisted of plasma cfDNA samples subjected to WGS from 231 patients with bile duct cancer (*n* = 25), breast cancer (*n* = 54), CRC (*n* = 27), duodenal cancer (*n* = 1), gastric cancer (*n* = 27), lung cancer (*n* = 35), ovarian cancer (*n* = 28) and pancreatic cancer (*n* = 34) and healthy individuals (*n* = 246) (dbGaP, Accession No. 34536)^10^.

The inhouse NSCLC dataset consisted plasma cfDNA samples subjected to whole-exome sequencing from 21 patients with NSCLC and 20 non-cancerous controls.

### Data preprocessing

We counted the frequencies of 256 4-kmers from the 5’-end of sequenced cfDNA reads.

We sorted the end-motifs by frequency in a descending order to obtain a sequence of end-motifs. The input to EMIT was formulated as ***M*** = {*CLS*, *m*_*0*_, *m*_*1*_, …, *m*_*i*_, …, *m*_*t*_, *SEP*; *t* < 256}, where *m*_*0*_, *m*_*1*_, …, *m*_*i*_ are end-motifs. The frequencies of these end-motifs are *m*_*0*_ ≥ *m*_*1*_ ≥ … ≥ *m*_*i*_ ≥ … ≥ *m*_*t*_. *t* is a predefined value and it was set to 128. *m*_*i*_ is an end-motif token. *CLS* and *SEP* are two specifical tokens added at the start and end of the sequence, respectively. To increase the datapoints, we generated the end-motif sequences for 10 million sequencing reads randomly without replacement.

### The architecture of end-motif Inspection via Transformer (EMIT)

EMIT is a transformer^18^ consisted of an encoder module and a projection head. The encoder module includes an embedding layer, a self-attention module followed by position-wise feed-forward network. The projection head is a two-layer neural network.

Embedding layer transforms the input end-motif tokens (parameterized by *W*_*x*_) and positions (parameterized by *W*_*p*_) into a representation matrix. Specifically, the embedding layer embeds the *i*th motif *m*_*i*_ into a feature vector of *d*-dimension *x*_*i*_, giving rise to a feature matrix of ***X*** = {*x*_*0*_, *x*_*1*_, …, *x*_*i*_…, *x*_*t*_; *t* < 4^*k*^}^T^ for an end-motif sequence ***M***. Besidies, the embedding layer embeds the positions of each end-motif in ***M*** into a feature vector of *d*-dimension, denoted as ***P*** = {*p*_*0*_, *p*_*1*_, …, *p*_*t*_; *t* < 4^*k*^}^T^. The transformer encoder takes the element-wise summation of ***X*** and ***P*** (i.e., ***X*** + ***P***) as input and outputs a representation matrix ***Z***.

The encoder layer is a transformer block that consists of multi-headed self-attention module followed by a *position-wise feed-forward neural network* (*FFN*). *Layer-wise normalization*^35^ is used in the front and rear of *FFN*. *Residual connection*^36^ is added to improve information flow.

Self-attention performs scale dot-product between *Q*, *K* and *V*^18^:

1 $$SelfAttn(Q,K,V)=soft{max }\left(\frac{Q{K}^{{\rm{T}}}}{\sqrt{{d}_{k}}}\right)V$$

Q, K and V are matrices projected from the outputs of the embedding layer. The scaling factor $\sqrt{{d}_{k}}$ is used to mitigate the extreme small gradient^18^.

Multi-headed self-attention allows the model to jointly attend to information from different representation subspaces at different positions, which is formulated as:

2 $$MultiHead(Q,K,V)=Concat(SelfAtt{n}_{1},{\mathrm{..}}.,SelfAtt{n}_{h})$$

where *h* denotes the number of attention heads.

Position-wise *FFN* is a fully-connected neural layer. The layer consists of two linear transformations with a *ReLU* activation function in between, which is defined as:

3 $$FFN(x)=\,{\max }(0,x{W}_{1}+{b}_{1}){W}_{2}+{b}_{2}$$

where *W*_*1*_ and *W*_*2*_ are weight matrices and ${b}_{1}$ and ${b}_{2}$ are the bias.

The output of the *Encoder* is fed into projection head. The projection head includes a two-layer neural network with layer-wise normalization^35^ in between.

### Different size of EMIT models

We trained three EMIT models that have different parameter size by varying the transformer hidden size of 384, 768 and 1536: EMIT-2Mb, EMIT-8Mb and EMIT-32Mb, giving rise to the number of attention heads of 6, 12 and 24, respectively. We used the exponentiated cross entropy (ECE) as the primary metric to compare these models.

### Development of EMIT

EMIT takes a sequence of end-motif tokens with a length of 128 as input. We hold out a random number of 5000 instances for evaluation. We followed the training scheme proposed by Devlin and colleagues^22^: the training data generator randomly corrupt 15% of each input motif sequence by replacing the chosen motif with [MASK] motif at 80%, random motif at 10% and the same motif at 10%. We trained these models by using Adam optimizer, batch size of 256, weight decay of 0.01 and learning rate of 1e−4 for 40 epochs. Learning rate was warmup for one epoch and decreased towards zero by following cosine scheduling. EMIT was trained with *PyTorch* (version 1.7.1) and transformers (version 4.21.1) on NVIDIA DGX A100 with 8 GPUs each with 40 Gb memory. Input sequence length was set to 128.

### Attentions among end-motifs

The attention between end-motif *i* and *j*, ${\alpha }_{{\boldsymbol{i}}{\boldsymbol{,}}{\boldsymbol{j}}}$, is defined as softmax-normalized dot product between the query and key vectors:

4 $${\rm{A}}=SelfAttn(Q,K,V)=soft{max}\left(\frac{Q{K}^{{\rm{T}}}}{\sqrt{{d}_{k}}}\right)V$$

5 $${\alpha }_{i,j}=A(i,j)$$

According to Clark and colleagues, the *CLS* token is used to aggregate representations of each end-motif to represent the input sequence^22^. Therefore, the attention weight of the *j*th end-motif on the *CLS* token, ${\alpha }_{CLS,j}$ represents the influence of that end-motif on the representation of the input sequence. The higher the value, the more important the end-motif on the representation of the sequence. We used Wilcoxon rank sum test^37^ to assess the difference of end-motifs in the patient group with cancer versus control group without cancer.

The end-motif attention matrix for cancer is obtained by taking the cumulative sum of ${\alpha }_{i,j}$ over all instances of cancer. We retained the top 0.1% interactions in subsequence analysis. The end-motif attention networks were visualized using *Cytoscape*^38^ (version 3.9.0).

### Linear projection of representations from EMIT

Linear projection in essence is a linear classifier that was used to determine whether the input representations are linear separable. Linear projection is widely used to determine whether specific features are encoded and represented within deep learning models^20,39,40^. We performed five-fold cross-validation to examine the linear projection accuracy for the collected public datasets. We examined sequence lengths of 64, 96, 128, 160, 200 and 256, respectively.

### Motif diversity score (MDS)

We calculate the motif diversity score according to Jiang and collegues^3^, which is defined as:

6 $${MDS}=\mathop{\sum }\limits_{i=1}^{256}-{P}_{i}* {\log (P}_{i})/\log (256)$$

Where *P*_*i*_ is the frequency of the ith motif.

### Baseline method

We considered linear projection of the count matrix of 4-kmer end-motifs as the baseline method for comparison.

### *kBET* test for assessment of batch effect

To assess the presence of batch effects, we conducted statistical analyses using the k-nearest neighbors batch effect test (*kBET*)^25^ on the Cristiano dataset. We used the volume value of plasma per sample as batch variable. Assumed the dataset has *n* samples, *m* batches and there are ${n}_{j}$ samples in *j*^th^ (*j* ≤ *m*) batch. The batch mixing frequency is denoted as $f=({f}_{1},\cdots ,{f}_{m})$, where ${f}_{j}=\frac{{n}_{j}}{N}$. The number of neighbors for the *i*th sample belonging to batch *j* is ${n}_{ji}^{k}$. The ${\chi }^{2}$ statistic is ${k}_{i}^{k}=\mathop{\sum }\limits_{j=1}^{m}\frac{{({n}_{ji}^{k}\,-\,{f}_{j}\cdot k)}^{2}}{{f}_{j}\cdot k}$ with a degree of freedom of *m* − 1. The *P* value is calculated according to ${p}_{i}^{k}=1-{F}_{m-1}({k}_{i}^{k})$, where ${F}_{m-1}(x)$ represents the cumulated density function. The *kBET* acceptance rate is defined as the percentage of samples that accept the null hypothesis at significance level α as follows:

7 $$kBET{\hbox{-}}rate=\frac{{\sum }_{i=1}^{N}I({p}_{i}^{k}\ge \alpha )}{N}\times 100 \%$$

where the indicator function *I(x)* = 1 if x > 0 otherwise *I(x)* = 0. We used *Pegasus* (version 1.4.3) to calculate the *kBET* acceptance rate by setting *K* and α to 5 and 0.05, respectively.

### Statistical analysis

We conducted our experiment with Python (version 3.7.10), R (version 4.2.1), ggplot2 (version 3.3.6) and PROC (version 1.18.0). Calculation of Area under the receiver operating characteristic curve (AUROC) was performed with PROC. The 95% confidence intervals of the AUROC were calculated using DeLong’s methods implemented in pROC. We calculated accuracy, sensitivity and specificity using R software package caret (version 6.0.78). The calculation of 95% confidence intervals for accuracy, sensitivity and specificity with Clopper−Pearson method^41^. Benjamini–Hochberg procedure was used to adjust *p*-values for multiple hypothesis test when appropriate. Two-sided test was used if not specified.

### Supplementary information

SUPPLEMENTAL MATERIAL

## Data Availability

Data is publicly available at European Bioinformatics Institute (No.: EGAS00001003409), Sequence Read Archive (No.: PRJNA574555 and PRJNA360288) and Genome Sequence Archive (No.: PRJCA000816 and PRJCA000646) databases.

## Code availability

Code will be available at https://github.com/deeplearningplus/EMIT.
