# Supplementary material for: Development of a deep learning model for cancer diagnosis by inspecting cell-free DNA end-motifs
Source: NPJ Precis Oncol. 2024 Jul 27;8:160. doi: 10.1038/s41698-024-00635-5 (PMC11283569; doi:10.1038/s41698-024-00635-5)
Supplement: Supplementary file 1 — SUPPLEMENTAL MATERIAL [file 41698_2024_635_MOESM1_ESM.pdf]

Supplementary Figures and Legends

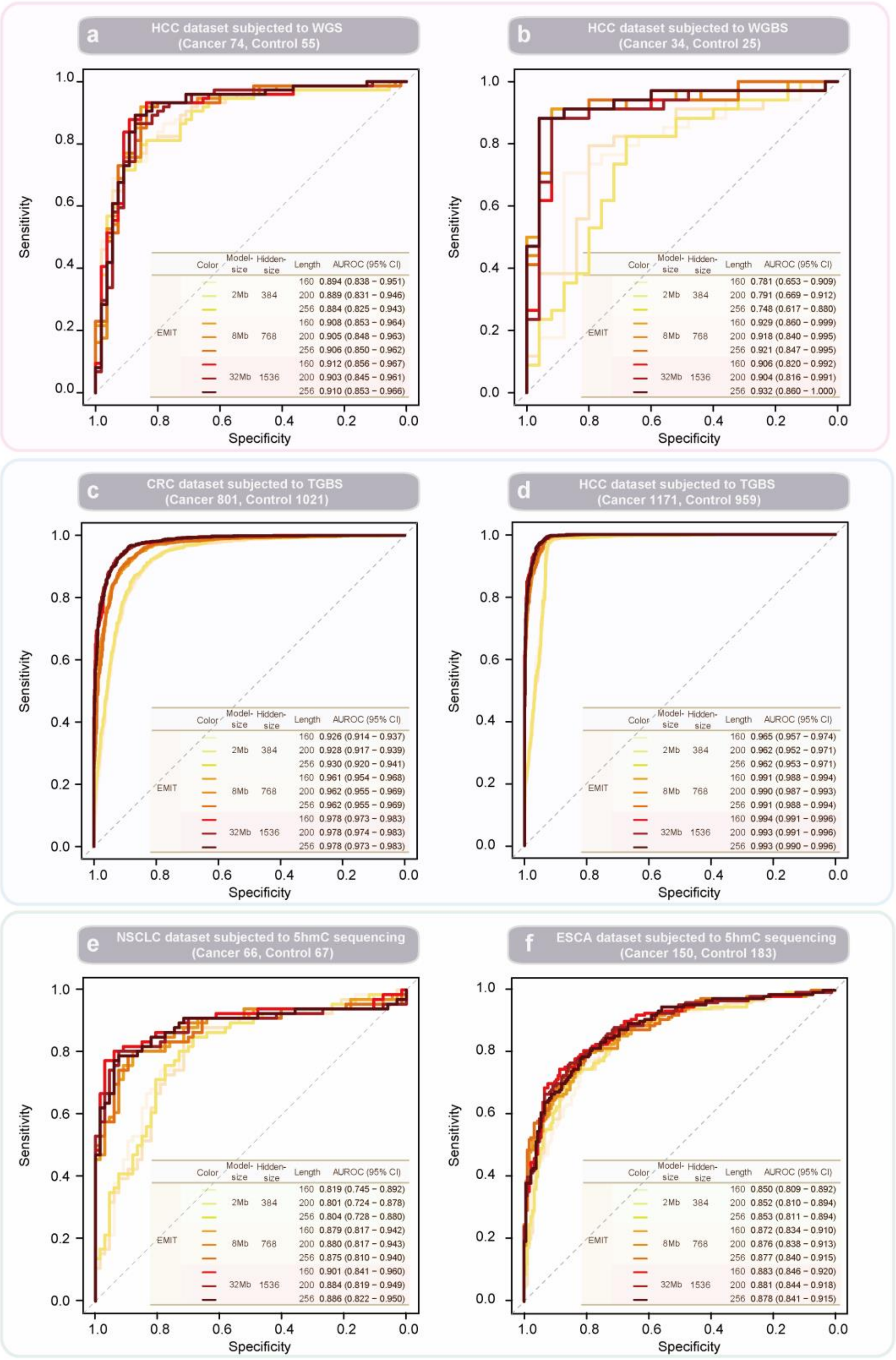

Supplementary Fig. 1 | ROC curves of different EMIT models with different number of motifs in identification of cancer across six datasets. a ROC curves on the hepatocellular carcinoma dataset subjected to whole-genome sequencing. **b** ROC curves on the hepatocellular carcinoma dataset subjected to whole-genome bisulfite

sequencing. **c** ROC curves on the colorectal dataset subjected to targeted bisulfite sequencing. **d** ROC curves on the hepatocellular carcinoma dataset subjected to targeted bisulfite sequencing. **e** ROC curves on the lung cancer dataset subjected to 5-hydroxymethylcytosine sequencing. **f** ROC curves on the esophageal carcinoma dataset subjected to 5-hydroxymethylcytosine sequencing.

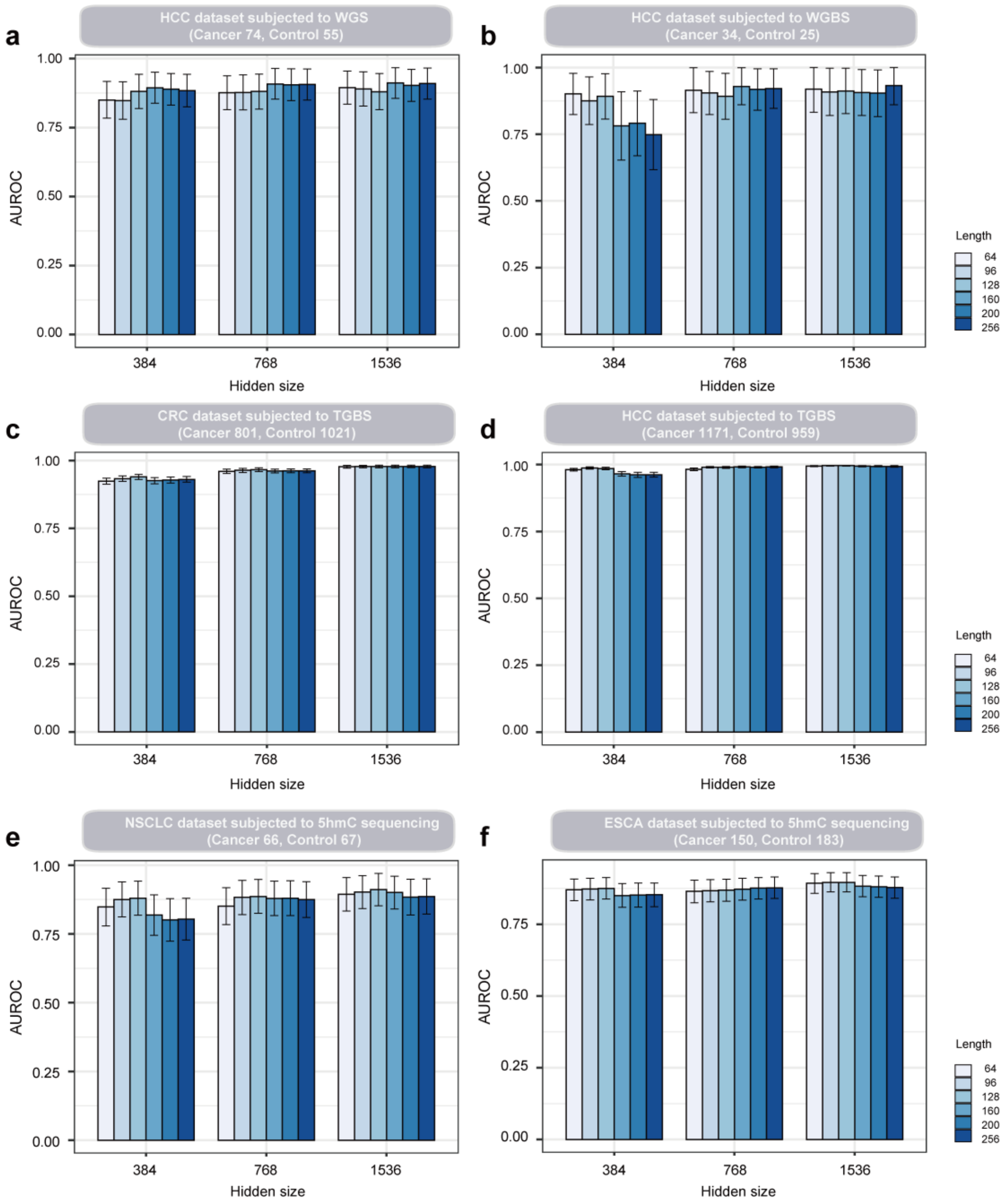

Supplementary Fig. 2 | The performance of EMIT with different number of end motifs across different model sizes on the six datasets. The bar represents the AUROC value, and the upper and lower 95% confidence intervals are shown as error bars.

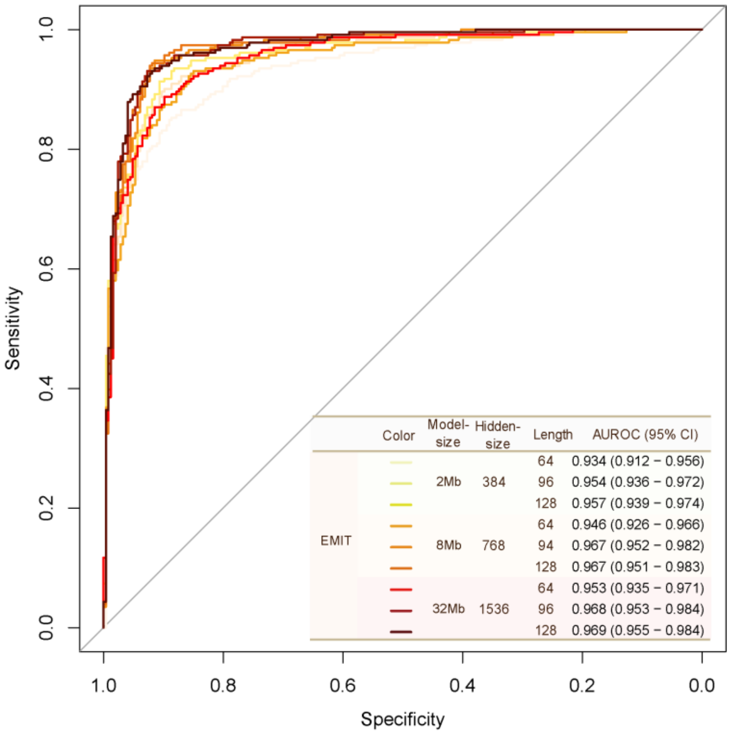

Supplementary Fig. 3 | Receiver operating characteristic curves of EMIT models on Cristiano dataset.

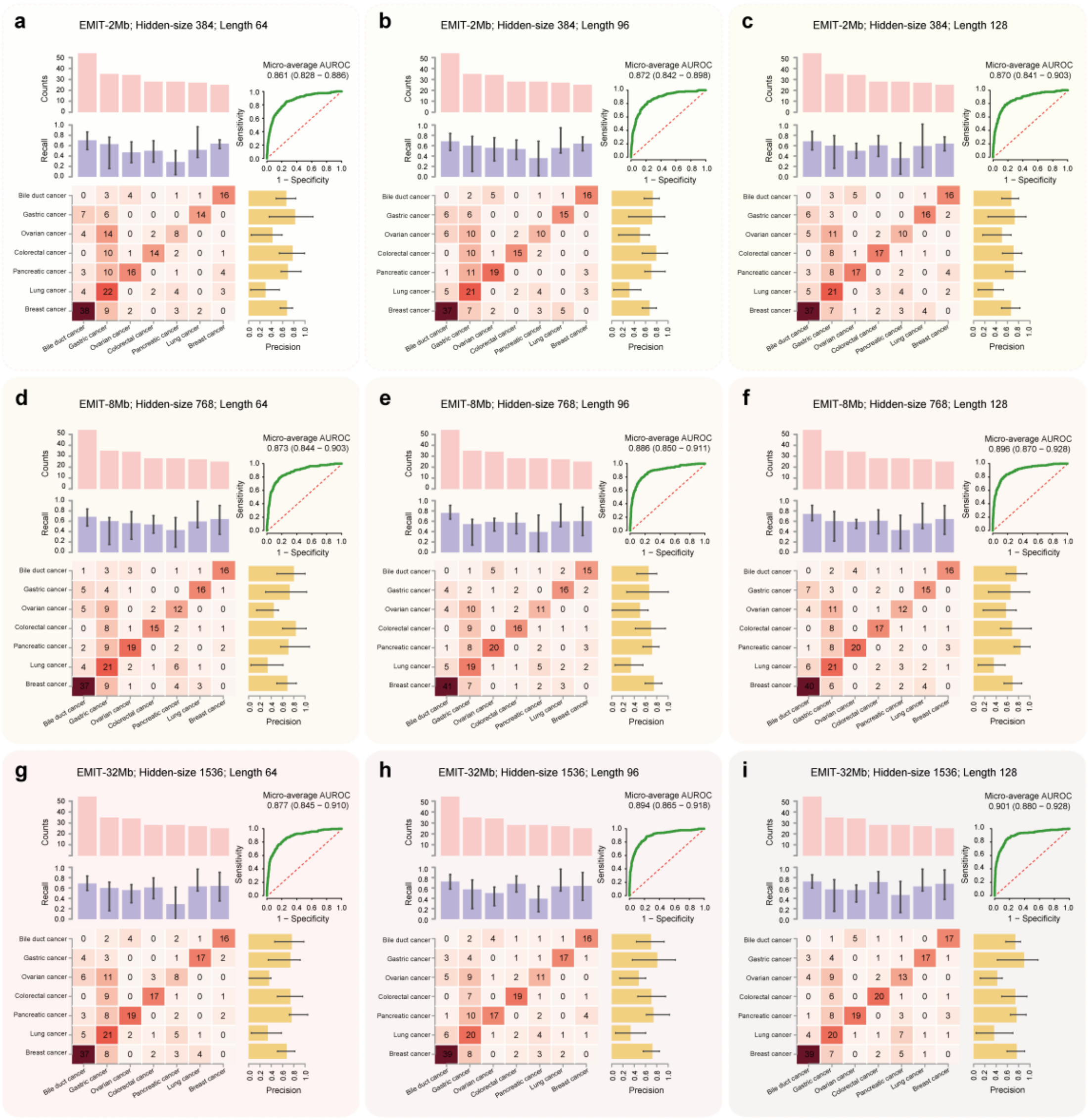

Supplementary Fig. 4 | The performance of EMIT in classification of multiple cancer types on the Cristiano dataset. Confusion matrix together with precision and recall are shown. Upper-right panel shows the micro-averaged one-versus-rest ROC curves.

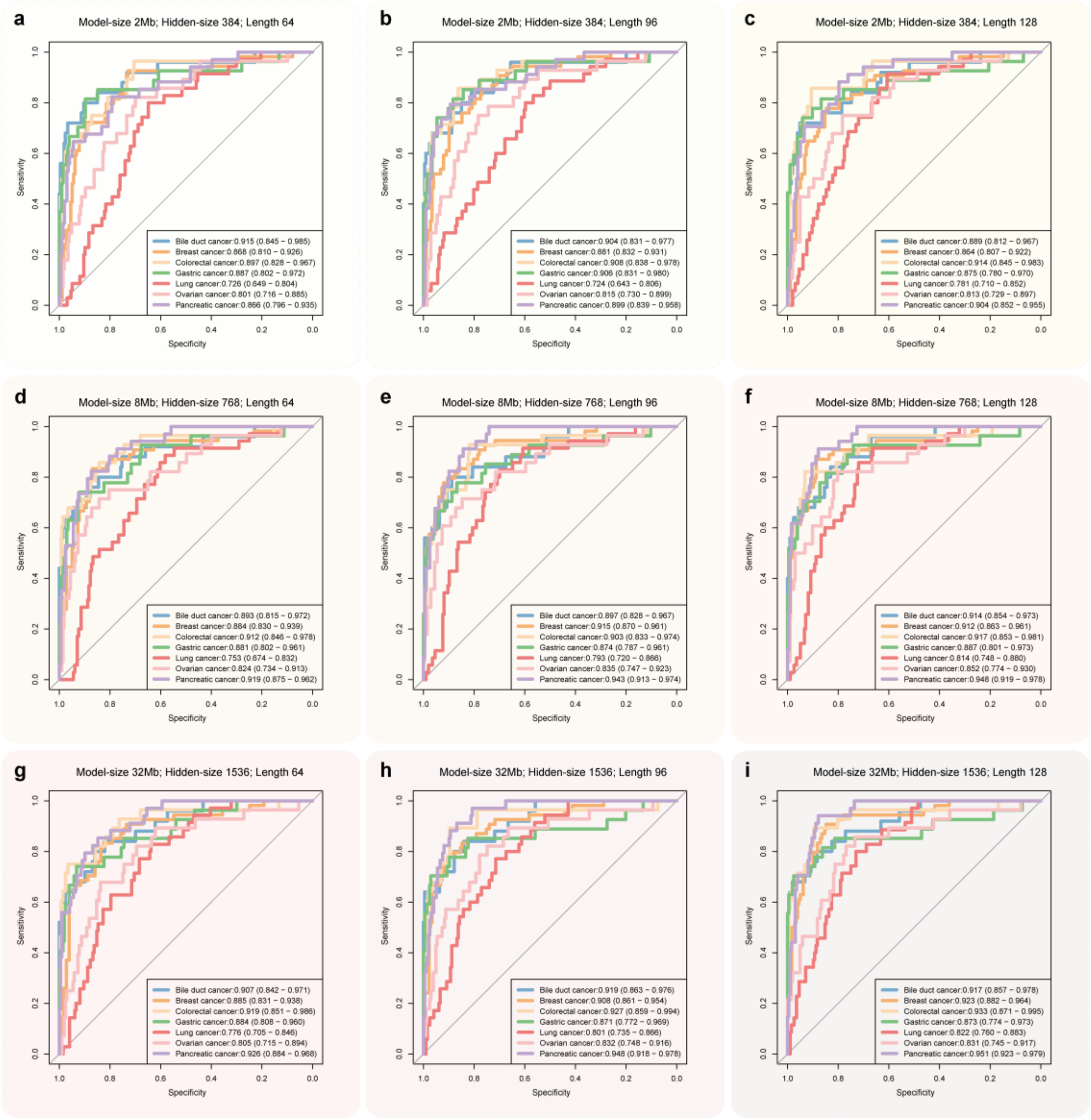

Supplementary Fig. 5 | ROC curves of EMIT stratified by cancer types on the Cristiano dataset.

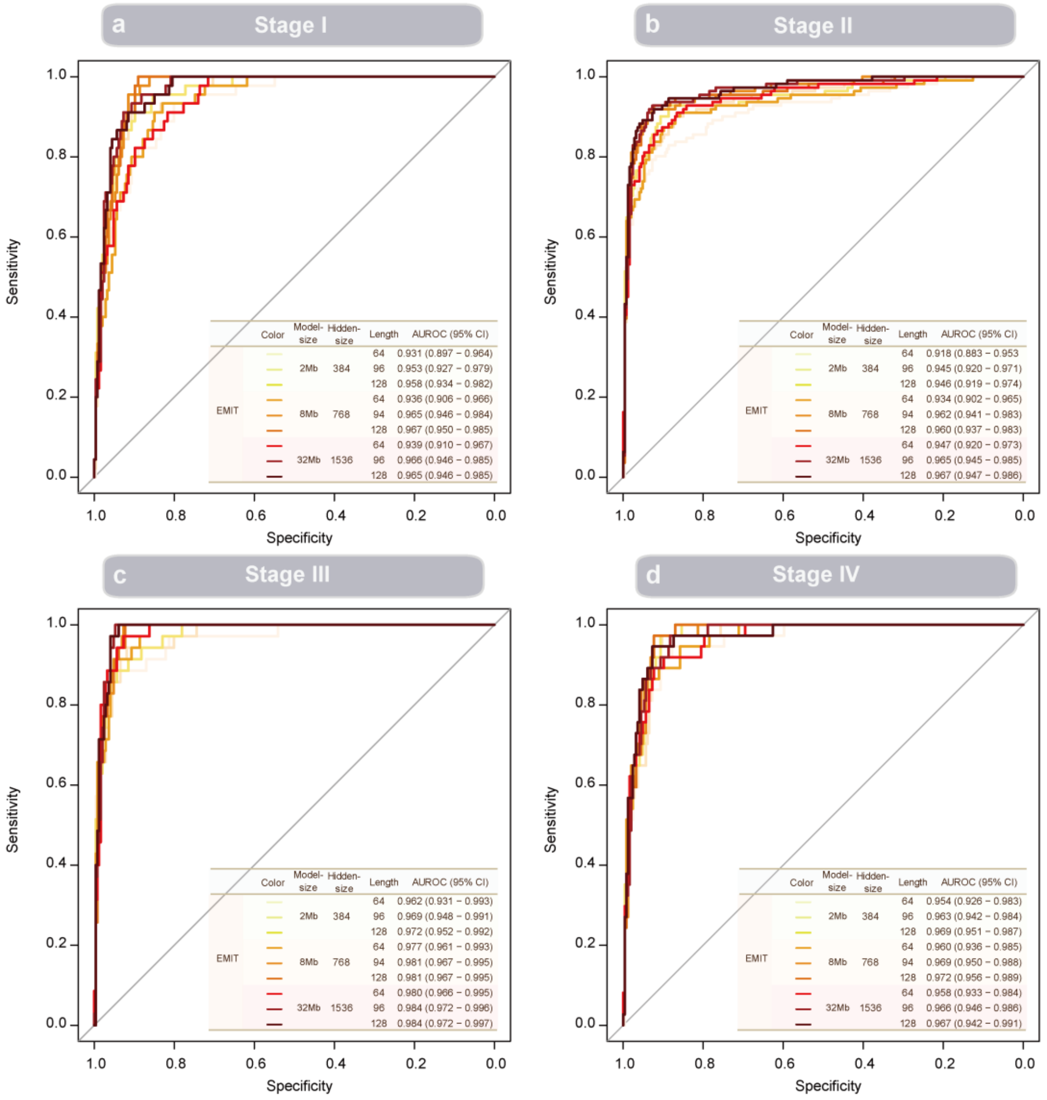

Supplementary Fig. 6 | Receiver operating characteristic curves of EMIT models stratified by tumor stages on Cristiano dataset.

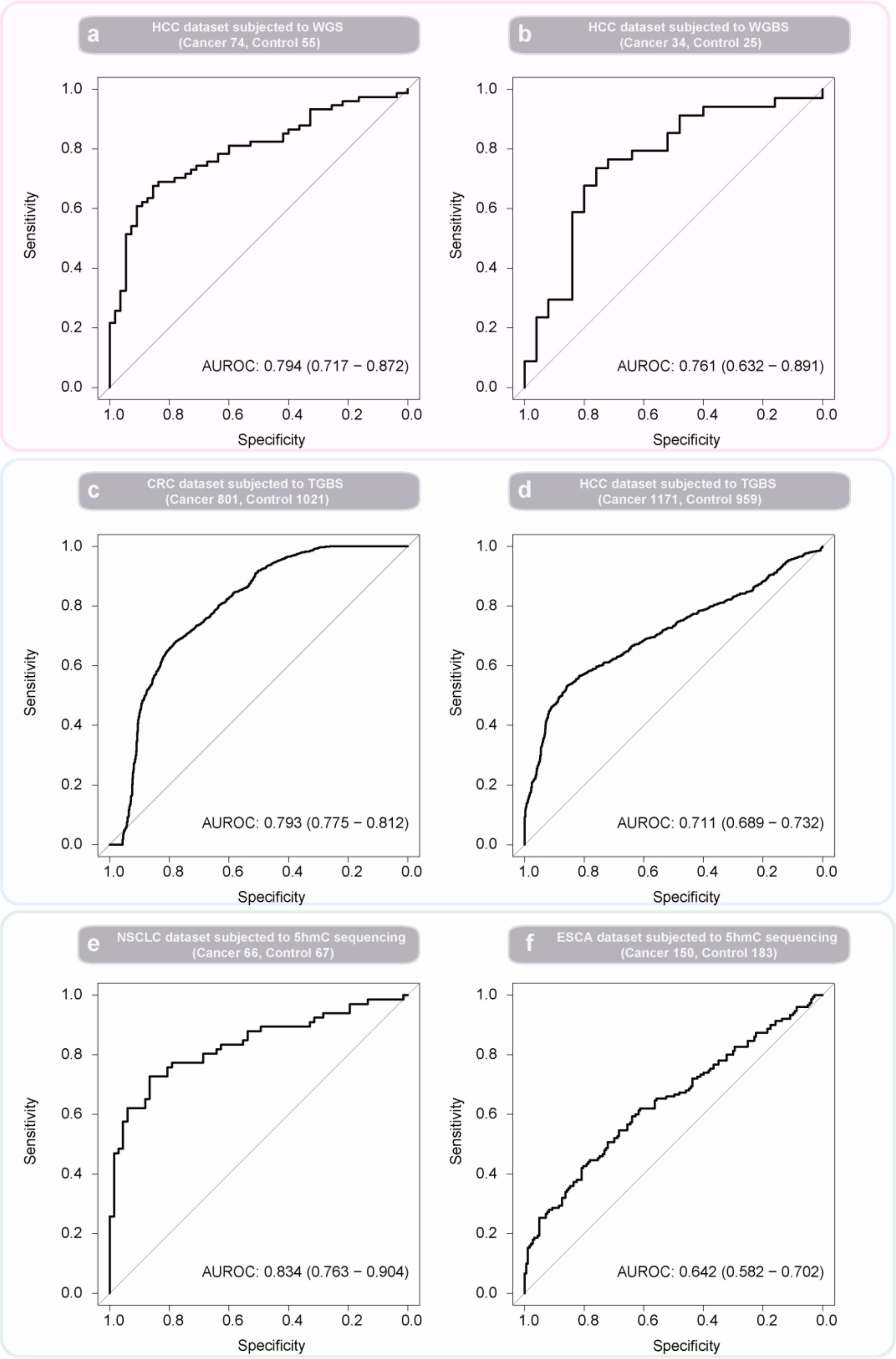

Supplementary Fig. 7 | The ROC curves of motif diversity score.

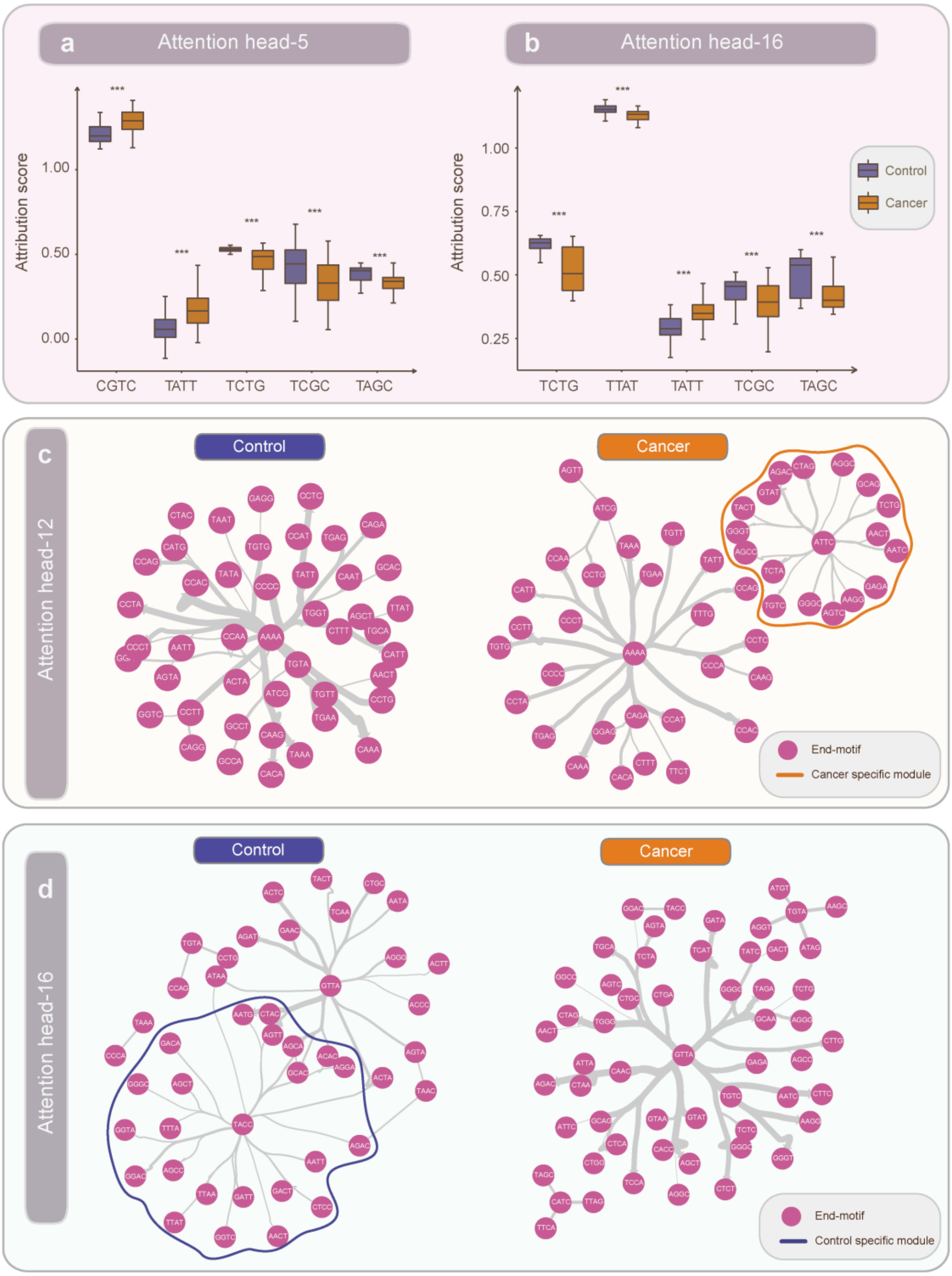

Supplementary Fig. 8 | End-motifs and end-motif attention netoworks exhibited differential patterns in cancer versus control. a and **b** End-motifs exhibited differential attribution scores. **c** and **d** End-motif attention netoworks exhibited differential topology.

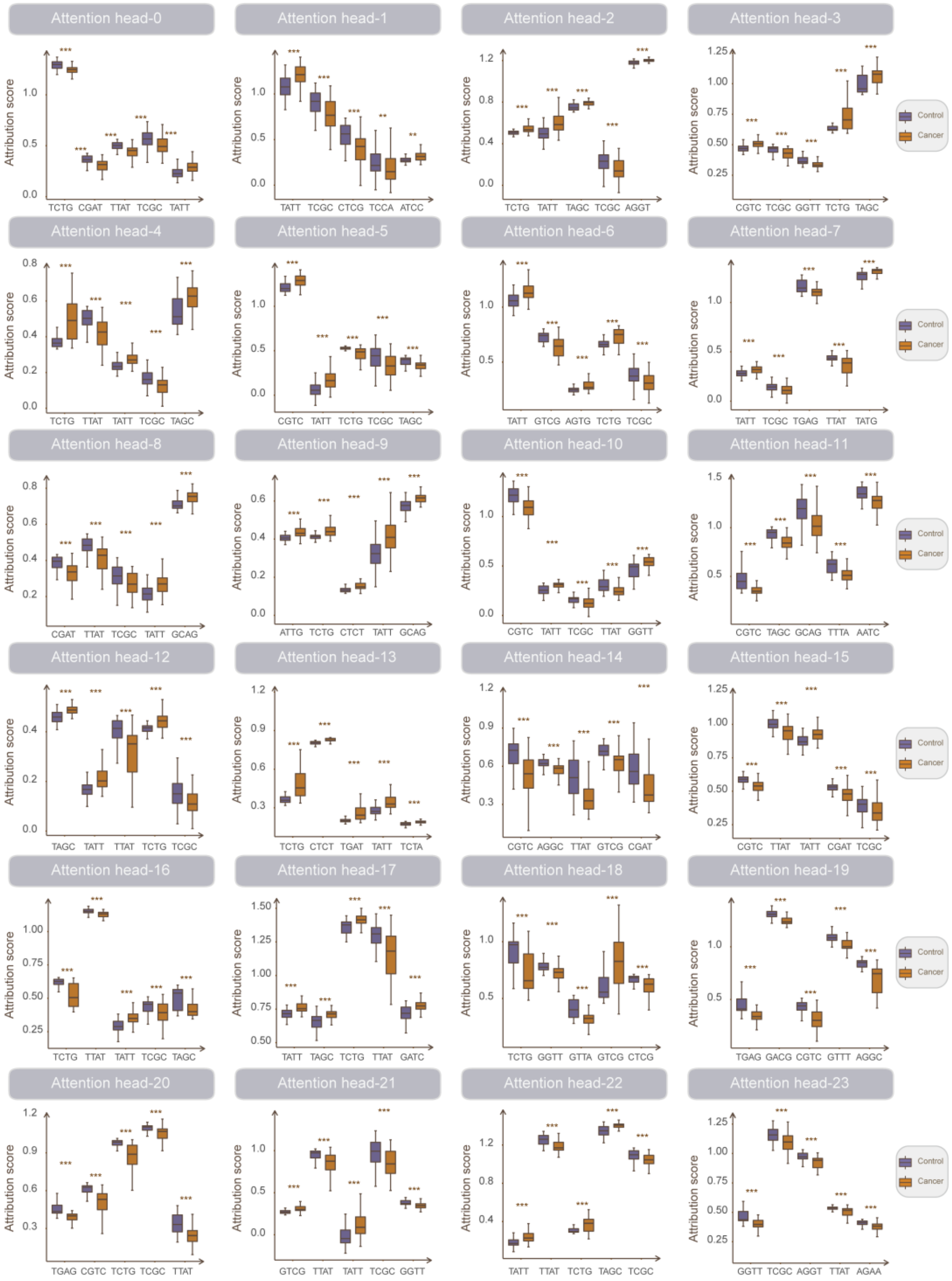

Supplementary Fig. 9 | Boxplots of attribution scores derived from 24 self-attention heads of EMIT-32Mb model for five representative end-motifs exhibiting significant difference in control group versus cancer group on the hepatocellular carcinoma dataset subjected to whole-genome sequencing. The boxplot represents the distribution of the attribution score, where the box spans the interquartile range (IQR) from the first quartile (Q1) to the third quartile (Q3). The median is denoted by the center line within the box, while the whiskers extend to encompass data points within 1.5 times the IQR from Q1 and Q3.

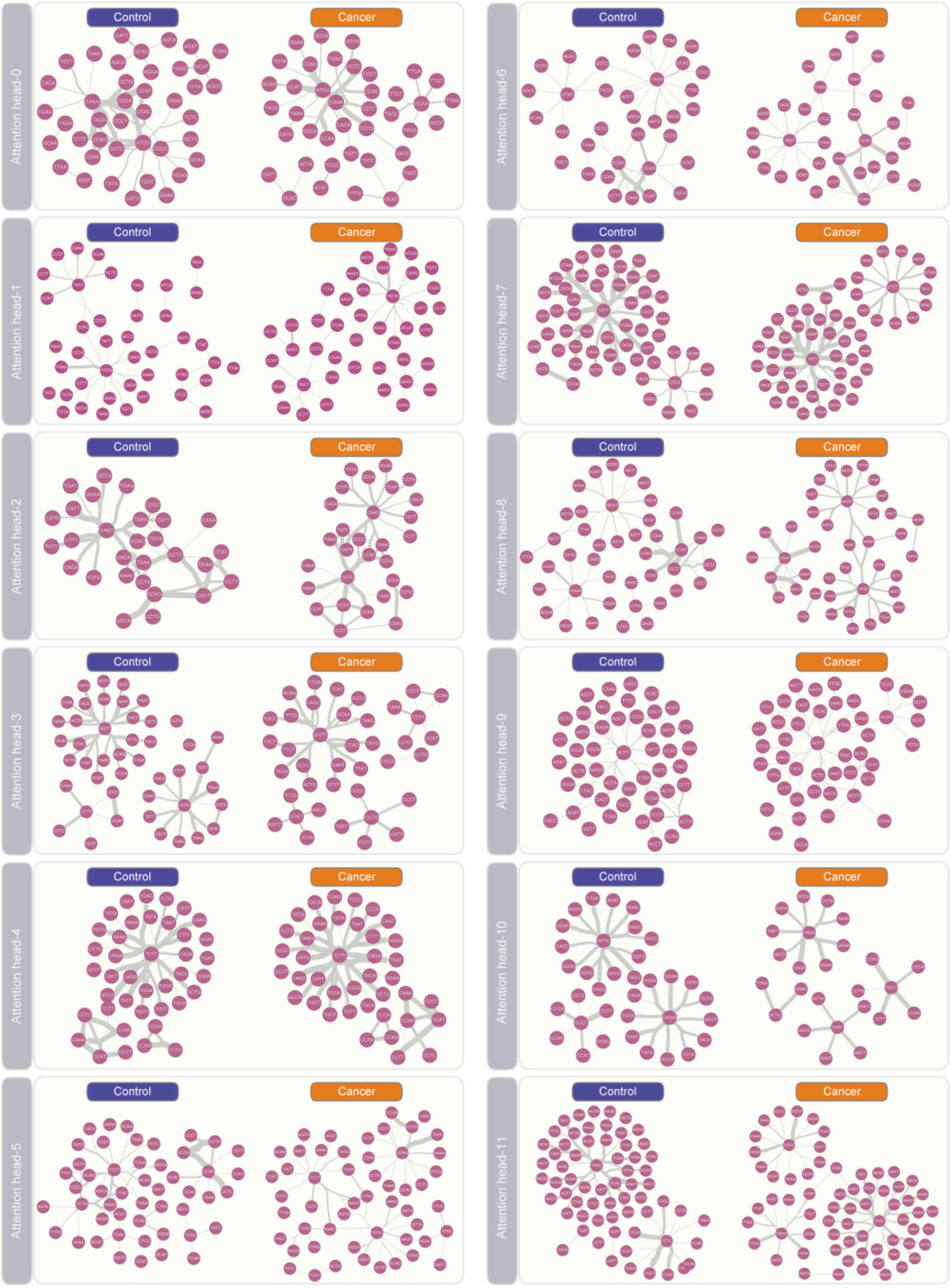

Continue to the next page

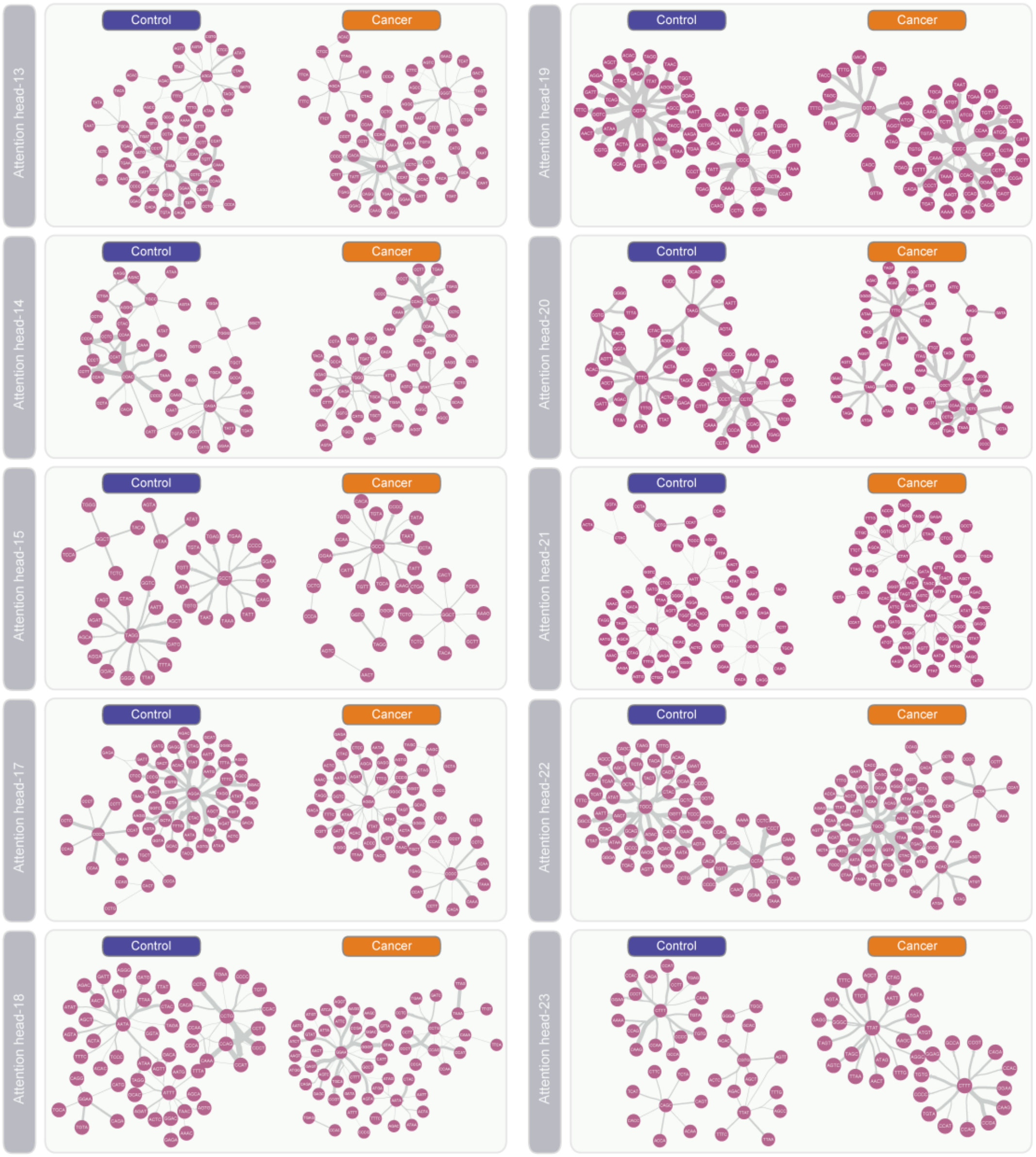

Supplementary Fig. 10 | End-motif networks derived from 24 self-attention heads of EMIT-32Mb model on the hepatocellular carcinoma dataset subjected to whole-genome sequencing. Thickness of edges represent attention scores.

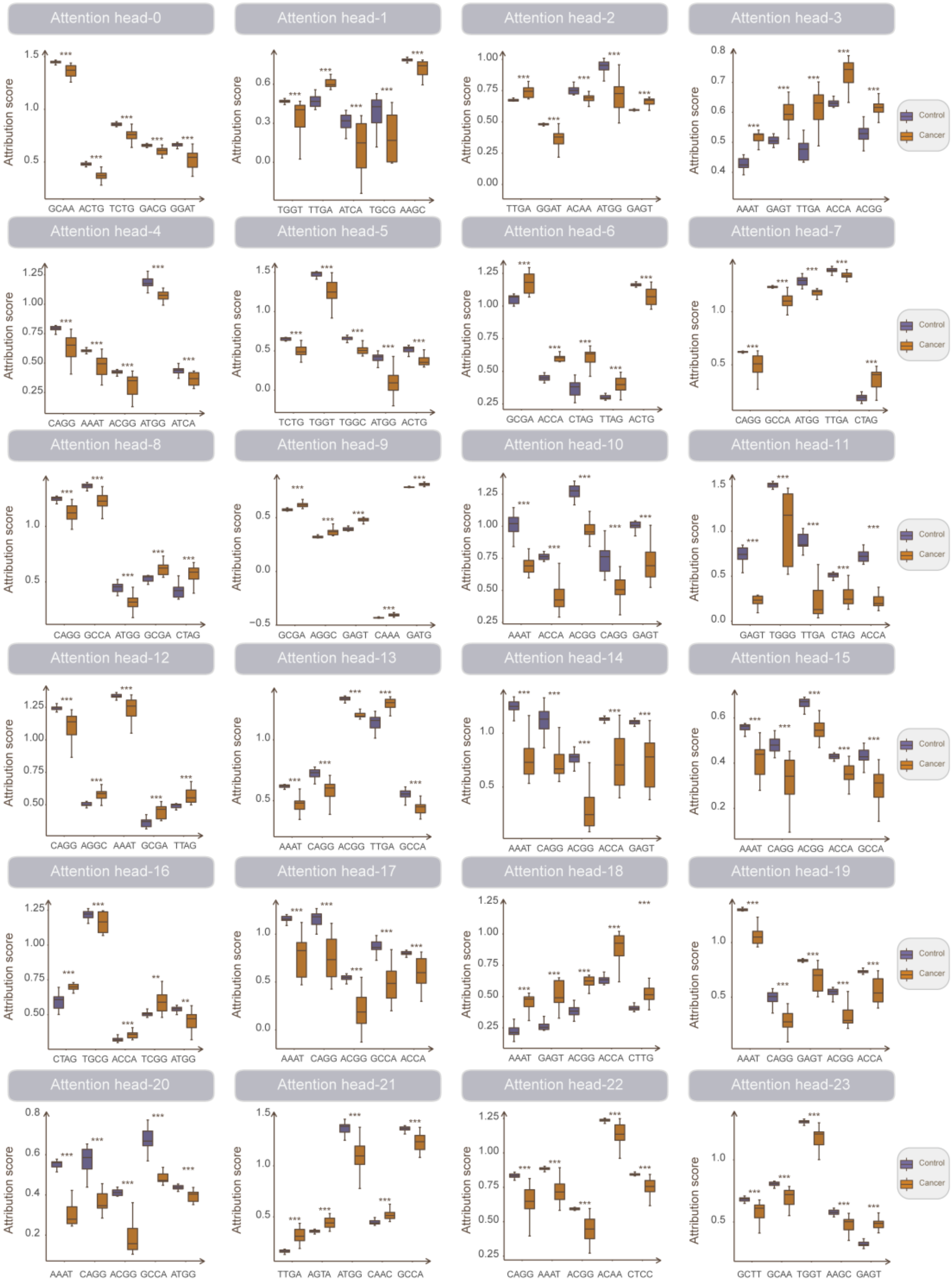

Supplementary Fig. 11 | Boxplots of attribution scores derived from 24 self-attention heads of EMIT-32Mb model for five representative end-motifs exhibiting significant difference in patients with lung cancer versus controls without lung cancer on the inhouse dataset subjected to whole-exome sequencing. The boxplot represents the distribution of the attribution score, where the box spans the interquartile range (IQR) from the first quartile (Q1) to the third quartile (Q3). The median is denoted by the center line within the box, while the whiskers extend to encompass data points within 1.5 times the IQR from Q1 and Q3.

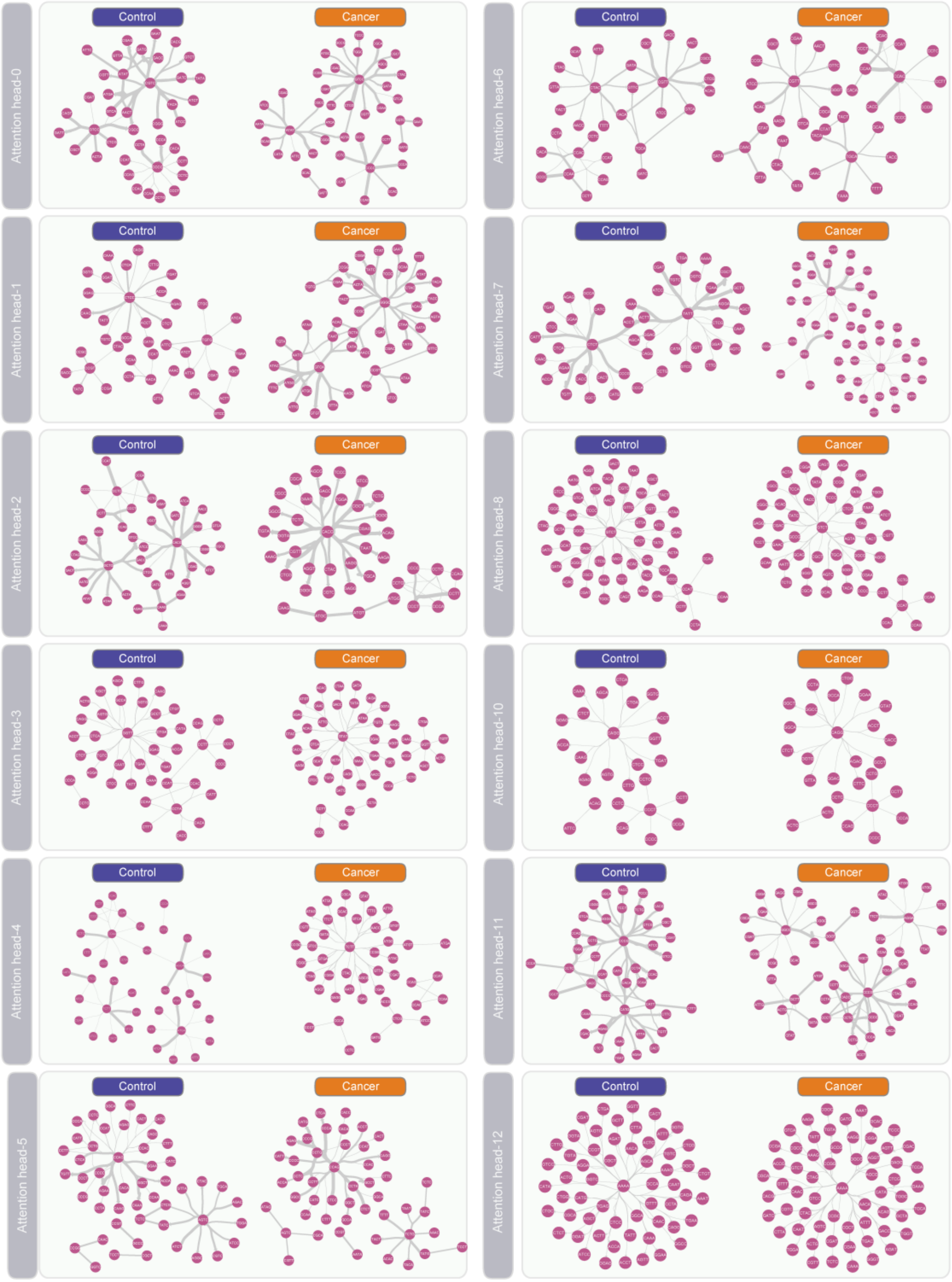

Continue to the next page.

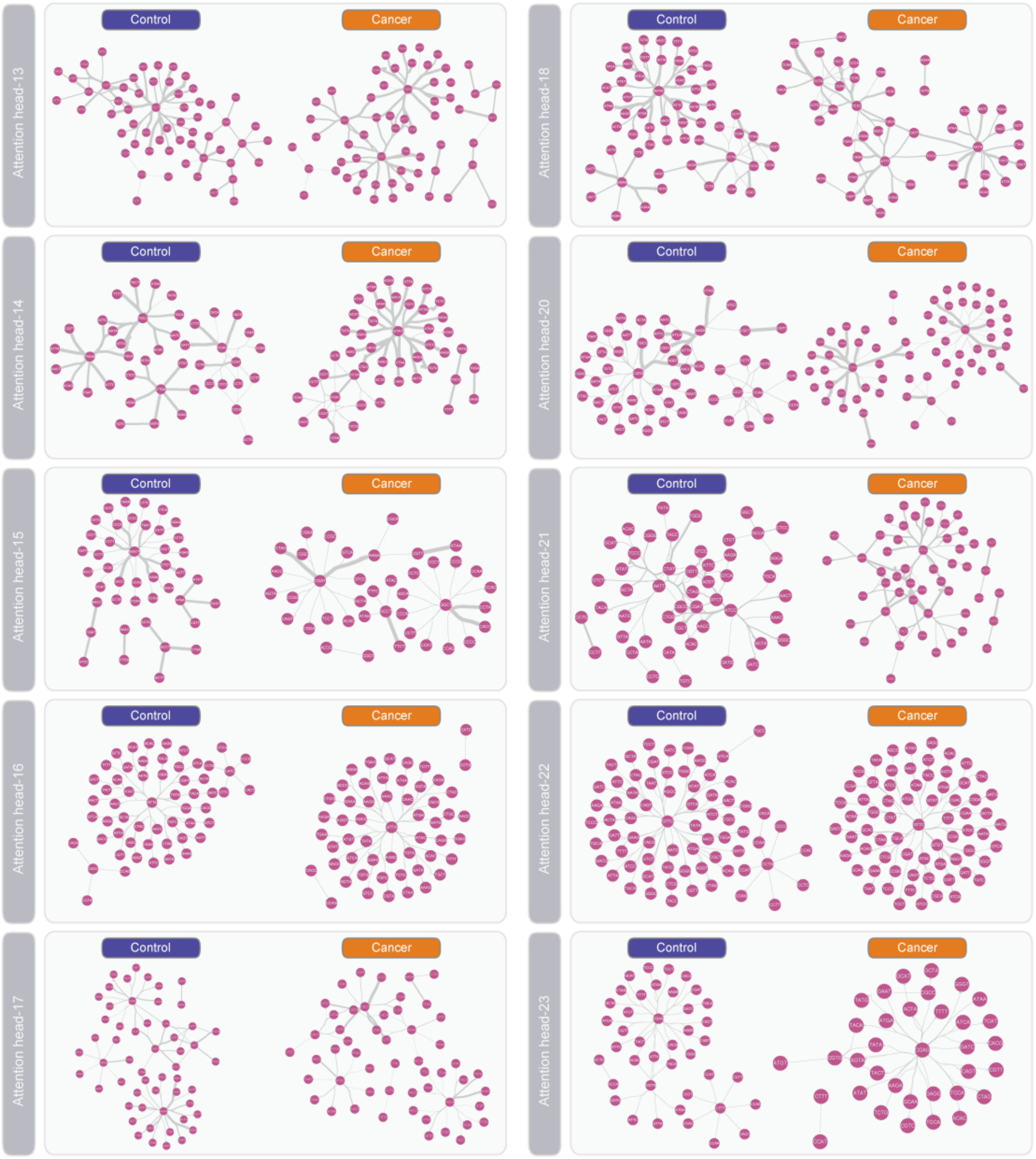

Supplementary Fig. 12 | End-motif networks derived from 24 self-attention heads of EMIT-32Mb model on the inhouse dataset subjected to whole-exome sequencing. Thickness of edges represent attention scores.

Supplementary Table 1. The AUROC value of EMIT with different number of end motifs across different model sizes on the six datasets.

	Length	HCC-WGS	HCC-WGBS	CRC-TGBS	HCC-TGBS	NSCLC-5hmC	ESCA-5hmC
EMIT-2Mb	64	0.850 (0.784 - 0.917)	0.901 (0.824 - 0.978)	0.924 (0.913 - 0.935)	0.981 (0.976 - 0.986)	0.848 (0.779 - 0.916)	0.870 (0.832 - 0.908)
	96	0.848 (0.780 - 0.916)	0.875 (0.786 - 0.965)	0.933 (0.923 - 0.943)	0.987 (0.983 - 0.991)	0.875 (0.812 - 0.939)	0.873 (0.835 - 0.910)
	128	0.881 (0.819 - 0.943)	0.892 (0.807 - 0.977)	0.940 (0.930 - 0.949)	0.985 (0.981 - 0.990)	0.880 (0.818 - 0.942)	0.875 (0.838 - 0.913)
	160	0.894 (0.838 - 0.951)	0.781 (0.653 - 0.909)	0.926 (0.914 - 0.937)	0.965 (0.957 - 0.974)	0.819 (0.745 - 0.892)	0.850 (0.809 - 0.892)
	200	0.889 (0.831 - 0.946)	0.791 (0.669 - 0.912)	0.928 (0.917 - 0.939)	0.962 (0.952 - 0.971)	0.801 (0.724 - 0.878)	0.852 (0.810 - 0.894)
	256	0.884 (0.825 - 0.943)	0.748 (0.617 - 0.880)	0.930 (0.920 - 0.941)	0.962 (0.953 - 0.971)	0.804 (0.728 - 0.880)	0.853 (0.811 - 0.894)
EMIT-8Mb	64	0.876 (0.815 - 0.938)	0.915 (0.831 - 0.999)	0.960 (0.952 - 0.968)	0.982 (0.977 - 0.987)	0.851 (0.784 - 0.918)	0.865 (0.825 - 0.904)
	96	0.877 (0.814 - 0.941)	0.905 (0.824 - 0.985)	0.964 (0.956 - 0.971)	0.990 (0.987 - 0.993)	0.883 (0.820 - 0.945)	0.867 (0.828 - 0.906)
	128	0.881 (0.817 - 0.944)	0.892 (0.806 - 0.978)	0.966 (0.959 - 0.973)	0.990 (0.986 - 0.993)	0.886 (0.825 - 0.948)	0.869 (0.830 - 0.907)
	160	0.908 (0.853 - 0.964)	0.929 (0.860 - 0.999)	0.961 (0.954 - 0.968)	0.991 (0.988 - 0.994)	0.879 (0.817 - 0.942)	0.872 (0.834 - 0.910)
	200	0.905 (0.848 - 0.963)	0.918 (0.840 - 0.995)	0.962 (0.955 - 0.969)	0.990 (0.987 - 0.993)	0.880 (0.817 - 0.943)	0.876 (0.838 - 0.913)
	256	0.906 (0.850 - 0.962)	0.921 (0.847 - 0.995)	0.962 (0.955 - 0.969)	0.991 (0.988 - 0.994)	0.875 (0.810 - 0.940)	0.877 (0.840 - 0.915)
EMIT-32Mb	64	0.895 (0.835 - 0.955)	0.919 (0.832 - 1.000)	0.977 (0.972 - 0.982)	0.994 (0.992 - 0.996)	0.894 (0.833 - 0.955)	0.893 (0.858 - 0.927)
	96	0.890 (0.828 - 0.952)	0.908 (0.820 - 0.997)	0.978 (0.973 - 0.982)	0.996 (0.994 - 0.997)	0.902 (0.842 - 0.962)	0.896 (0.863 - 0.930)
	128	0.880 (0.815 - 0.946)	0.912 (0.827 - 0.997)	0.978 (0.973 - 0.983)	0.996 (0.994 - 0.997)	0.911 (0.852 - 0.970)	0.896 (0.863 - 0.930)
	160	0.912 (0.856 - 0.967)	0.906 (0.820 - 0.992)	0.978 (0.973 - 0.983)	0.994 (0.991 - 0.996)	0.901 (0.841 - 0.960)	0.883 (0.846 - 0.920)
	200	0.903 (0.845 - 0.961)	0.904 (0.816 - 0.991)	0.978 (0.974 - 0.983)	0.993 (0.991 - 0.996)	0.884 (0.819 - 0.949)	0.881 (0.844 - 0.918)
	256	0.910 (0.853 - 0.966)	0.932 (0.860 - 1.000)	0.978 (0.973 - 0.983)	0.993 (0.990 - 0.996)	0.886 (0.822 - 0.950)	0.878 (0.841 - 0.915)

Supplementary Table 2. The classification metrics of linear projections of EMIT models on hepatocellular carcinoma dataset subjected to whole-genome sequencing.

Input feature	Model-size	Hidde-size	Length	Accuracy (95% CI)	Sensitivity (95% CI)	Specificity (95% CI)
Motif count	-	-	-	0.698 (0.611 - 0.775)	0.667 (0.568 - 0.756)	0.833 (0.626 - 0.953)
Representation from EMIT model	2Mb	384	64	0.783 (0.702 - 0.851)	0.774 (0.670 - 0.858)	0.800 (0.654 - 0.904)
		384	96	0.791 (0.710 - 0.857)	0.805 (0.699 - 0.887)	0.769 (0.632 - 0.875)
		384	128	0.829 (0.753 - 0.890)	0.833 (0.732 - 0.908)	0.824 (0.691 - 0.916)
	8Mb	768	64	0.806 (0.727 - 0.870)	0.802 (0.699 - 0.883)	0.812 (0.674 - 0.911)
		768	96	0.837 (0.762 - 0.896)	0.853 (0.753 - 0.924)	0.815 (0.686 - 0.907)
		768	128	0.845 (0.771 - 0.903)	0.855 (0.756 - 0.925)	0.830 (0.702 - 0.919)
	32Mb	1536	64	0.845 (0.771 - 0.903)	0.865 (0.765 - 0.933)	0.818 (0.691 - 0.909)
		1536	96	0.845 (0.771 - 0.903)	0.875 (0.776 - 0.941)	0.807 (0.681 - 0.900)
		1536	128	0.845 (0.771 - 0.903)	0.855 (0.756 - 0.925)	0.830 (0.702 - 0.919)

Supplementary Table 3. The classification metrics of linear projections of EMIT models on hepatocellular carcinoma dataset subjected to whole-genome bisulfite sequencing.

Input feature	Mode l size	Hidden size	Length	Accuracy (95% CI)	Sensitivity (95% CI)	Specificity (95% CI)
Motif count	-	-	-	0.695 (0.561 - 0.808)	0.667 (0.516 - 0.796)	0.818 (0.482 - 0.977)
Representation from EMIT model	2Mb	384	64	0.831 (0.710 - 0.916)	0.875 (0.710 - 0.965)	0.778 (0.577 - 0.914)
		384	96	0.814 (0.691 - 0.903)	0.848 (0.681 - 0.949)	0.769 (0.564 - 0.910)
		384	128	0.831 (0.710 - 0.916)	0.833 (0.672 - 0.936)	0.826 (0.612 - 0.950)
	8Mb	768	64	0.881 (0.771 - 0.951)	0.909 (0.757 - 0.981)	0.846 (0.651 - 0.956)
		768	96	0.881 (0.771 - 0.951)	0.935 (0.786 - 0.992)	0.821 (0.631 - 0.939)
		768	128	0.831 (0.710 - 0.916)	0.900 (0.735 - 0.979)	0.759 (0.565 - 0.897)
	32Mb	1536	64	0.915 (0.813 - 0.972)	0.939 (0.798 - 0.993)	0.885 (0.698 - 0.976)
		1536	96	0.915 (0.813 - 0.972)	0.939 (0.798 - 0.993)	0.885 (0.698 - 0.976)
		1536	128	0.915 (0.813 - 0.972)	0.939 (0.798 - 0.993)	0.885 (0.698 - 0.976)

Supplementary Table 4. The classification metrics of linear projections of EMIT models on colorectal cancer dataset subjected to targeted bisulfite sequencing.

Input feature	Model-size	Hidde -size	Length	Accuracy (95% CI)	Sensitivity (95% CI)	Specificity (95% CI)
Motif count	-	-	-	Accuracy (95% CI)	SN (95% CI)	SP (95% CI)
Representation from EMIT model	2Mb	384	64	0.757 (0.736 - 0.776)	0.819 (0.784 - 0.850)	0.729 (0.704 - 0.754)
		384	96	0.856 (0.842 - 0.870)	0.921 (0.906 - 0.934)	0.777 (0.752 - 0.801)
		384	128	0.863 (0.849 - 0.876)	0.920 (0.905 - 0.933)	0.791 (0.766 - 0.814)
	8Mb	768	64	0.879 (0.866 - 0.892)	0.933 (0.919 - 0.946)	0.811 (0.787 - 0.833)
		768	96	0.919 (0.908 - 0.929)	0.937 (0.924 - 0.949)	0.892 (0.871 - 0.910)
		768	128	0.920 (0.909 - 0.930)	0.936 (0.922 - 0.947)	0.896 (0.875 - 0.914)
	32Mb	1536	64	0.916 (0.905 - 0.926)	0.936 (0.922 - 0.947)	0.886 (0.866 - 0.905)
		1536	96	0.926 (0.916 - 0.936)	0.936 (0.923 - 0.947)	0.912 (0.892 - 0.928)
		1536	128	0.923 (0.912 - 0.933)	0.944 (0.932 - 0.955)	0.892 (0.872 - 0.910)

Supplementary Table 5. The classification metrics of linear projections of EMIT models on hepatocellular carcinoma dataset subjected to targeted bisulfite sequencing.

Input feature	Model-size	Hidden-size	Length	Accuracy (95% CI)	Sensitivity (95% CI)	Specificity (95% CI)
Motif count	-	-	-	0.944 (0.933 - 0.953)	0.913 (0.897 - 0.928)	0.988 (0.979 - 0.994)
Representation from EMIT model	2Mb	384	64	0.946 (0.936 - 0.956)	0.927 (0.911 - 0.941)	0.973 (0.960 - 0.983)
		384	96	0.957 (0.947 - 0.965)	0.945 (0.930 - 0.957)	0.973 (0.960 - 0.982)
		384	128	0.954 (0.945 - 0.963)	0.937 (0.922 - 0.950)	0.978 (0.966 - 0.986)
	8Mb	768	64	0.946 (0.936 - 0.956)	0.927 (0.911 - 0.941)	0.973 (0.960 - 0.983)
		768	96	0.954 (0.944 - 0.962)	0.933 (0.918 - 0.946)	0.982 (0.971 - 0.990)
		768	128	0.958 (0.948 - 0.966)	0.938 (0.923 - 0.950)	0.985 (0.975 - 0.992)
	32Mb	1536	64	0.964 (0.956 - 0.972)	0.944 (0.930 - 0.956)	0.992 (0.984 - 0.997)
		1536	96	0.969 (0.961 - 0.976)	0.955 (0.942 - 0.966)	0.987 (0.977 - 0.993)
		1536	128	0.971 (0.963 - 0.978)	0.959 (0.946 - 0.969)	0.988 (0.979 - 0.994)

Supplementary Table 6. The classification metrics of linear projections of EMIT models on lung cancer dataset subjected to 5-hydroxymethylcytosine sequencing.

Input feature	Model-size	Hidden-size	Length	Accuracy (95% CI)	Sensitivity (95% CI)	Specificity (95% CI)
Motif count	-	-	-	0.714 (0.630 - 0.789)	0.792 (0.650 - 0.895)	0.671 (0.560 - 0.769)
Representation from EMIT model	2Mb	384	64	0.782 (0.702 - 0.849)	0.794 (0.673 - 0.885)	0.771 (0.656 - 0.863)
		384	96	0.820 (0.744 - 0.881)	0.862 (0.746 - 0.939)	0.787 (0.677 - 0.873)
		384	128	0.812 (0.735 - 0.875)	0.806 (0.691 - 0.892)	0.818 (0.704 - 0.902)
	8Mb	768	64	0.774 (0.694 - 0.842)	0.781 (0.660 - 0.875)	0.768 (0.651 - 0.861)
		768	96	0.820 (0.744 - 0.881)	0.850 (0.734 - 0.929)	0.795 (0.684 - 0.880)
		768	128	0.850 (0.777 - 0.906)	0.859 (0.750 - 0.934)	0.841 (0.733 - 0.918)
	32Mb	1536	64	0.850 (0.777 - 0.906)	0.859 (0.750 - 0.934)	0.841 (0.733 - 0.918)
		1536	96	0.865 (0.795 - 0.918)	0.914 (0.810 - 0.971)	0.827 (0.722 - 0.904)
		1536	128	0.872 (0.803 - 0.924)	0.945 (0.849 - 0.989)	0.821 (0.717 - 0.898)

Supplementary Table 7. The classification metrics of linear projections of EMIT models on esophageal carcinoma dataset subjected to 5-hydroxymethylcytosine sequencing.

Input feature	Model-size	Hidden-size	Length	Accuracy (95% CI)	Sensitivity (95% CI)	Specificity (95% CI)
Motif count	-	-	-	0.748 (0.698 - 0.794)	0.766 (0.682 - 0.837)	0.737 (0.672 - 0.795)
Representation from EMIT model	2Mb	384	64	0.802 (0.755 - 0.843)	0.784 (0.709 - 0.847)	0.816 (0.753 - 0.869)
		384	96	0.808 (0.761 - 0.849)	0.836 (0.760 - 0.895)	0.790 (0.728 - 0.844)
		384	128	0.775 (0.726 - 0.819)	0.766 (0.687 - 0.833)	0.781 (0.716 - 0.838)
	8Mb	768	64	0.778 (0.729 - 0.821)	0.775 (0.697 - 0.842)	0.779 (0.715 - 0.836)
		768	96	0.793 (0.745 - 0.835)	0.814 (0.736 - 0.877)	0.779 (0.716 - 0.834)
		768	128	0.796 (0.748 - 0.838)	0.825 (0.748 - 0.887)	0.778 (0.715 - 0.832)
	32Mb	1536	64	0.811 (0.765 - 0.851)	0.813 (0.738 - 0.874)	0.809 (0.747 - 0.862)
		1536	96	0.808 (0.761 - 0.849)	0.847 (0.771 - 0.905)	0.785 (0.723 - 0.838)
		1536	128	0.814 (0.768 - 0.854)	0.861 (0.786 - 0.917)	0.787 (0.725 - 0.840)
